# Tumor Metabolism as a Regulator of Tumor–Host Interactions in the B-Cell Lymphoma Microenvironment—Fueling Progression and Novel Brakes for Therapy

**DOI:** 10.3390/ijms20174158

**Published:** 2019-08-26

**Authors:** Anna C. Beielstein, Christian P. Pallasch

**Affiliations:** Department I of Internal Medicine, Center for Integrated Oncology Aachen Bonn Cologne Duesseldorf, CECAD Center of Excellence on Cellular Stress Responses in Aging-Associated Diseases, Center for Molecular Medicine Cologne (CMMC), University of Cologne, Josef Stelzmann Street 24, 50937 Cologne, Germany

**Keywords:** Lymphoma, metabolism, glycolysis, lactate, IDO, microenvironment, macrophage, T-cell, PD-L1

## Abstract

Tumor metabolism and its specific alterations have become an integral part of understanding functional alterations leading to malignant transformation and maintaining cancer progression. Here, we review the metabolic changes in B-cell neoplasia, focusing on the effects of tumor metabolism on the tumor microenvironment (TME). Particularly, innate and adaptive immune responses are regulated by metabolites in the TME such as lactate. With steadily increasing therapeutic options implicating or utilizing the TME, it has become essential to address the metabolic alterations in B-cell malignancy for therapeutic approaches. In this review, we discuss metabolic alterations of B-cell lymphoma, consequences for currently used therapy regimens, and novel approaches specifically targeting metabolism in the TME.

## 1. Metabolism in Cancer—Resurrection of an Ancient Finding

Malignant transformation of cells leads to numerous specific alterations defining the neoplastic characteristics which have been comprehensively summarized as the major “hallmarks of cancer” [1]. However, in the initial version of this seminal approach to summarize carcinogenesis, both aspects of tumor metabolism, the specific involvement of the tumor microenvironment and immune modulatory features, were not yet included. Further progress inevitably led to the identification of both aspects as important hallmarks [2]. The rapidly evolving field of tumor metabolism research has yielded numerous important insights into the specific alterations and dependencies of metabolism in malignant cells. The various dimensions have been in turn comprehensively summarized as “hallmarks of tumor metabolism” by Pavlova and Thompson [3].

The work on cancer metabolism has come back into the focus of tumor biology after almost 75 years since the discovery of the ”Warburg Effect“—the shift of aerobic to anaerobic glycolysis in malignant tumors [4]. More recently, the aberrant expression of the pyruvate kinase M2 isoform has been described to underlie this so far understudied phenomenon. The shift of PKM1 towards PKM2 functionally determines a preferential anaerobic glycolysis leading to metabolism of glucose to lactate and a far less efficient generation of ATP. Several functional implications for this shift have been discussed and the improved shift towards NADPH generation and subsequent feed of anabolic pathways, such as lipogenesis, have primarily been discussed [5].

Another recent prominent example of metabolism-associated genes being discovered for functional implication in malignant transformations is the mutation of the isocitrate dehydrogenase 1 and 2 (IDH1/IDH2) in gliomas and acute myeloid leukemia [6]. These mutations change enzymatic properties, producing 2-hydroxyglutarate (2HG) from α-ketoglutarate and subsequently inhibiting cell differentiation by inhibition of histone demethylation [7].

Assessment of metabolic activity has been a widely utilized feature in diagnostics of malignant disease—FDG-PET scans display glucose metabolism as a surrogate marker for malignant cell activity. In Hodgkin’s lymphoma, it has become essential for upfront diagnostics as well as for assessment of treatment response [8]. Particularly, in Hodgkin’s lymphoma, PET diagnostics have gained an established role despite the fact that, in this specific entity, the amount of tumor cells is highly variable and represents only a minor proportion of the tumor tissue. This, however, indicates the relevance of assessing the metabolic alterations from a microenvironment perspective. Nonmalignant bystander cells have to be considered as major contributors to metabolism and the functional status of tumor tissue.

In parallel to the field of tumor metabolism, the perception of the tumor microenvironment in cancer has undergone an even more prominent development, most prominently demonstrated by the eruption of novel immunotherapies using checkpoint inhibitors in steadily increasing number of entities including B-cell lymphomas [9,10,11,12,13]. In B-cell lymphoma, the contribution of the tumor microenvironment to disease progression has been clearly established as important for immune therapies, checkpoint inhibitors, and chemo-immunotherapies [9,14].

In this review, we attempt to shed light on the specific perturbations of tumor metabolism in the microenvironment of B-cell malignancies that alter both the biological functions of malignant lymphoma as well as their non-transformed counterparts within the microenvironment. These alterations inherently harbor therapeutic relevance, both for currently utilized approaches as well as for future concepts and agents.

## 2. Metabolic Alterations in B-Cell Malignancies

Cellular metabolism in B-cell lymphoma and leukemias can be affected on several functional levels ranging from genomic aberrations to post-translational lipid modifications. A prominent example of tumor metabolism driver mutations was first identified in glioma and acute myeloid leukemia (AML). In 20% of AML cases, a mutation in isocitrate dehydrogenase (IDH) 1 or 2 can be detected [15,16]. These mutations occur as an early event in the pathogenesis of AML and are already evident in preleukemic hematopoietic stem cells [17]. IDH catalyzes the decarboxylation of isocitrate to α-ketoglutarate and CO_2_, IDH1 in the cytosol, and IDH2 in the mitochondria. Therefore, IDH plays an important role in cellular redox state regulation and the defense against oxidative stress [18,19,20]. Upon mutation, IDH discontinues to synthesize α-ketoglutarate and switches towards generation of the ‘oncometabolite’ 2-hydroxyglutarate (2-HG) [21]. Accumulation of 2-HG in the leukemic stem cells leads to DNA and histone hypermethylation, which leads to global dysregulation of gene expression, a block of myeloid cell differentiation, and the promotion of leukemogenesis [21,22]. The mutation of IDH1 leads to metabolic changes such as a decreased NADPH pool and impaired TCA cycle during cellular hypoxia [23,24]. The reduction of α-ketoglutarate due to mutated IDH indirectly influences other metabolic pathways, as a decrease of α-ketoglutarate correlates with increased expression of HIF1α [25].

In the attempt to identify classic driver mutations in B cell malignancy such as IDH in AML or glioma, recent sequencing approaches have not revealed similarly prominent metabolism genes as direct driver mutations of lymphomas [26,27]. However, alterations involving the MYC oncogene, such as translocations or overexpression of MYC, are a hallmark of B cell lymphoma pathogenesis [28]. The MYC oncogene alteration profoundly reprograms cellular metabolism as recently reviewed by Dejure et al. [29]. In brief, malignant cells require elevated MYC levels to sustain the high proliferative rate of lymphoma cells. Cells need to modulate MYC function and adapt to the availability of nutrients to avoid metabolic collapse. Particularly, nucleoside metabolism genes, as a prerequisite for proliferation and cell growth, are upregulated by MYC [30,31]. The glutamine metabolism is a key pathway regulated by MYC expression both altering glutamine uptake as well as glutaminolysis [29,32,33]. Additionally, MYC regulates glucose uptake and glycolysis as well as lipid biosynthesis [34,35,36]. MYC expression may also induce metabolic liabilities; as such, ornithine decarboxylase has been shown to be essential for EµM [37]. Tumor cells overexpressing MYC, such as non-Hodgkin lymphoma (NHL) cells, rely on glutamine metabolism as a fuel for the TCA cycle in the tumor microenvironment with its specific nutrient supply [38].

Another hallmark of B-cell malignancy is the altered B-cell receptor (BCR) signaling axis, which is crucial for maintenance and production of healthy and malignant B-cells. Tonic activation of this pathway was described in lymphoma and leukemia cells [39]. One downstream signaling branch of the BCR signaling is the PI3K/AKT/mTORC1 axis. This axis activates cellular prosurvival factors, but also has an impact on metabolic processes in B-cells. The PI3K isoform PI3Kα influences glycolysis and energy production, and subsequent AKT signaling affects the cellular metabolome [40,41,42]. AKT increases the expression and translocation of the glucose transporter GLUT1 and the expression of glycolytic enzymes, especially the expression, activation, and mitochondrial interaction of hexokinase (HK), thereby promoting glucose uptake and glycolysis [43]. By genetic inhibition of the BCR pathway or PI3K inhibition, a decrease in oxidative phosphorylation (OxPhos) and glycolysis was observed [40].

The AKT/mTORC1 signaling pathway was detected to be aberrantly activated also without the direct activation via BCR signaling in different B-cell malignancies [44]. As such, PTEN mutations occur in a subset of diffuse large B-cell lymphoma (DLBCL) and mantle cell lymphoma (MCL) and lead to expression of genes involved in the AKT/mTORC1 pathway [45,46]. These PTEN mutations are correlated with reduced overall survival upon chemotherapeutical R-CHOP treatment in DLBCL [45,47]. In follicular lymphoma, RagC mutations also reinforce mTORC1 signaling by abolishing the dependence on amino acids for mTORC1 activation [48]. Beside amino acids, mTORC1 could also be activated by glutamine, whose uptake is upregulated in several B-cell malignancies [49]. mTORC1 activity promotes several anabolic and energy-producing processes such as protein synthesis, pyrimidine synthesis, HIF1α expression, glycolysis, the oxidative part of the pentose phosphate pathway (PPP), lipid and mitochondrial metabolism, and glutaminolysis [50,51,52,53,54,55,56]. HIF1α and MYC activate the expression of genes encoding for glucose transporter (GLUT), hexokinase (HK), monocarboxylate transporter (MTC), pyruvate dehydrogenase (PDK), phosphofructokinase (PFK), phosphoglycerate kinase (PGK), pyruvate kinase (PK), and lactate dehydrogenase (LDHA), thereby strongly promoting anaerobic glycolysis [36,57,58]. Along these lines, the translation of cyclin D1 mRNA, which is required for cell cycle progression, is regulated by mTORC1 signaling [59]. These metabolic adaptations allow cells to highly proliferate and promote survival, as proliferation is impaired in mTORC1 knock-out DLBCL cell lines [60]. Inhibition of the PI3K/AKT/mTORC1 axis and inhibition of MYC lead to a decreased viability of lymphoma cells and a decreased glycolytic activity seen by reduced glucose uptake, glucose metabolism, and glycolytic gene expression [36].

Loss of the tumor suppressor PP2A (serine/threonine protein phosphatase 2A) is another origin of metabolic alteration in DLBCL, which leads to an increase in glycolysis [61].

In CLL cells, glycolysis is active; however, it does not reach similar levels as DLBCL lymphoma cells [62]. Instead, CLL cells use altered lipid metabolism through constitutive STAT3 activation, thereby promoting mitochondrial activity [63]. Increased mitochondrial activity in turn leads to increased levels of reactive oxygen species, which promote tumor progression by influencing the tumor microenvironment and facilitating apoptotic cell death [64,65]. When the metabolism of CLL changes cells switch to a glycolytic phenotype, seen by high FDG uptake in the PET-CT, this may be indicative of Richter´s transformation towards an aggressive lymphoma, mostly DLBCL [66]. Similarly, high glucose uptake is observed in Hodgkin lymphoma by PET diagnostics [8]. Here, it is noteworthy that Hodgkin lymphoma cells express high levels glucose transporter 1 (GLUT1) and lactate dehydrogenase (LDHA) [67].

In conclusion, numerous molecular mechanisms of altered metabolism have been identified in B-cell malignancy. While these alterations, such as MYC and the B-cell receptor PI3K/AKT axis, have been intensively studied, other pathways remain to be elucidated.

## 3. The Tumor Microenvironment as a Target of Tumor Metabolism

Metabolic alterations in leukemia and lymphoma are primarily seen as a result of alterations within the transformed cell clone. However, metabolism-based interactions of transformed and non-transformed cells within the tumor microenvironment have been increasingly observed as defining specific functional alterations.

### 3.1. Glycolytic Alteration Changes Macrophage Function

As described in the previous section, B-cell malignancies produce a significant proportion of ATP by anaerobic glycolysis. It was shown that in anaerobic glycolysis, the M2 isoform of pyruvate kinase (PKM2) is preferentially active and, in line with this observation, that PKM2 was the dominant isoform in variant tumor cell lines [68,69]. PKM2 leads to a higher production of lactate and pyruvate in the cells than the other isoform, PKM1, and the amount of lactate in tumor cell line media correlated with the tumor malignancy [69]. The acidification of the tumor microenvironment has been shown to have a direct impact on the polarization of infiltrating macrophages and drives them into a tumor-supporting subtype called ‘tumor-associated macrophages’ (TAMs). Microenvironmental acidification is sensed by GPCRs of macrophages and leads through an increase of cAMP by adenylyl cyclase activation to expression of the cAMP-responsive element modulator isoform ICER [70]. ICER expression inhibits the NF-κB signaling pathway, which is crucial for proinflammatory polarization of macrophages. This mechanism also downregulates proinflammatory TNF expression and induces genes associated with a noninflammatory macrophage subtype (i.e., Arg1, Clec10a, VEGF, HIF1α) [70,71,72]. The expression of vascular endothelial growth factor (VEGF), which causes neovascularization and arginase-1 (Arg1) induction, is also supported by the transcription factor HIF1α [69,73]. HIF1α itself is stabilized by lactate, hypoxia or IL-4, which are all present in the tumor microenvironment [69]. In total, TAMs have a higher expression of VEGF and Arg1 as compared to the other cellular components of the tumor microenvironment, which underlines the tumor-supporting function of TAMs [69]. Furthermore, HIF1α promotes expression of programmed death ligand-1 (PD-L1) on myeloid cells, which leads to expression of anti-inflammatory interleukin 10 (IL-10) [74].

Lactate-exposed TAMs express higher levels of glutamine synthetase, transaminase GPT, and enzymes of the urea cycle, contributing to nitrogen metabolism alterations in the tumor [69].

Additionally, TAMs produce the macrophage migration inhibitory factor (MIF), which suppresses TP53 transcription in tumor cells, thereby supporting the lack of DNA-damage-repair response and the accumulation of mutations in the tumor cells [75,76].

The prognostic value of high glycolytic activity was shown to correlate with a decreased success of checkpoint-inhibitor therapy and that the expression of ICER negatively correlates with ‘complete response’ [70,77,78,79].

### 3.2. Glycolytic Alteration Changes Cancer-Associated Fibroblast Function

In view of B-cell malignancies, acidification is also used as a tumor supporting feature. Cancer-associated fibroblasts (CAFs) from patients with DLBCL or follicular lymphoma show a highly increased secretion of pyruvate. Pyruvate secreted from CAFs corresponds to metabolic activity of lymphoma cells in coculture [80]. The co-cultivated lymphoma cells showed increased anaerobic glycolysis, indicated by increased expression of hexokinase 2 (HK2) and pyruvate dehydrogenase kinase (PDK). Additionally, an increased tricarboxylic acid cycle (TCA) and lower production of reactive oxygen species (ROS) was detected and assumed to be responsible for lymphoma cell survival [80,81]. Moreover, by adding pyruvate to culture media, survival of primary DLBCL cells was increased and lymphoma cells showed a higher resistance to doxorubicin after cultivating them in CAF-derived media [80].

In non-Hodgkin lymphomas (NHL), higher glycolytic activity is associated with higher aggressiveness, which is implemented through an interaction between the lymphoma cells and their surrounding microenvironment [82]. A higher expression of the lactate transporter monocarboxylate transporter 1 (MCT1) in NHL is associated with poor clinicopathological profile and the expression of MCT1 and MCT4 is associated with high-grade disease [82]. MCT1 expression may be regulated by MYC as MCT1 was significantly upregulated in c-MYC amplified lymphomas, also correlating with poor prognosis and survival [83,84]. The MTCs are the basis for the ‘lactate shuttle’, in which lactate is transferred between different cell types and can be utilized at the receiving end for energy production, thereby promoting proliferation and chemoresistance [85]. In DLBCL, nontransformed cells in the microenvironment upregulate MCT4. Thereby, they acquire the increased ability to export lactate. Corresponding upregulation of MCT1 on malignant cells subsequently increases import of lactate, which, in turn, is used to fuel the TCA cycle for energy production [82,85]. A similar change of expression profile was found in Hodgkin lymphoma, where transformed cells showed high expression of MCT1, and TAMs of MCT4 [86]. Beside the transport of lactate, MCT1 and MCT4 also interact with CD147 [87]. CD147 is associated with tumor aggressiveness and chemoresistance and its silencing leads to less proliferation and increased chemosensitivity in hematological malignancies [88,89,90]. Interruption of MCT1 activity by AZD3965 inhibited NHL cell viability, led to intracellular accumulation of lactate, and increased apoptotic cell death [82]. This effect could be further potentiated by adding metformin, which augments glycolysis in the cells, to the treatment [83]. The overexpression of MCT1 could be used as a therapeutic target also, as it was shown that overexpression of MCT1 sensitizes cells in vitro and in vivo to 3-bromopyruvic acid [84].

### 3.3. Glycolytic Alteration Changes T-Cell Function

In B-cell leukemia, other glycolysis-driven mechanisms of immunosuppression were observed. The leukemic B-cells cause T-cells to become hyporesponsive and metabolically compromised [91]. In CLL-derived T-cells, an impaired induction of activation markers CD25 and CD71 and impaired induction of glucose uptake and glycolysis was observed, while in BCR/ABL^+^ B cell lymphoblastic leukemia-derived T-cells, glucose uptake and expression of GLUT1 and HK2 was impaired after in vitro stimulation [91]. Resting T-cells dominantly use oxidative metabolism, but under activation induce GLUT1, HK2, and aerobic glycolysis [92]. For this induction of glucose uptake and glycolysis, the mTORC1 pathway plays a crucial role [93,94]. Chronic stimulation of T-cells induces expression of inhibitory receptors such as PD-1 or CD200 on T-cells, which impair upregulation of mTORC1 signaling and glucose uptake upon restimulation [91]. PD-1 ligation inhibits PI3K/Akt/mTOR signaling and MYC expression, thereby leading to decreased glycolysis and an increase of lipid oxidation [95,96,97]. The inhibition of glycolysis leads to an exhausted phenotype of T-cells and further PD-1 expression, thereby promoting tumor growth and impairing the outcome in B-cell malignancy [98,99,100,101]. Promoting this immunosuppressive effect, it was shown that leukemic and stromal cells increased expression of PD-L1 and Gal-9, which induces proliferation stop and cell death in T-cells [102,103]. As T_Reg_-cells rely on oxidative metabolism, they are unaffected by glycolysis inhibition and can still promote immunosuppression [104,105].

To counteract glycolysis inhibition via anti-PD-1, an increase in GLUT1 expression on CD8^+^ T-cells from leukemia-bearing mice was seen [91]. Moreover, the activation of the AKT/mTORC1 signaling decreased expression of PD-1 and TIM-3 and improved antitumor immunity in a mouse model [91].

As another mechanism of impeding T-cell activation, it was shown that the mitochondrial biogenesis and fitness of CD8^+^ T-cells is impaired in CLL patients and the reserve of GLUT1 is decreased [106]. During activation of physiological T-cells, mitochondrial mass increases to fuel increased metabolic needs and to stimulate the first step of glycolysis [107,108,109]. In CD8^+^ T-cells from CLL patients, no increase in mitochondrial mass was found after T-cell stimulation [106]. Moreover, less mitochondrial mass correlated with a higher expression of the inhibitory receptors PD-1, TIM-3, and LAG-3 [110].

In this context, it is noteworthy that in CAR-T-cell therapy of CLL, just a minor subset of patients showed a good response. It was observed that the mitochondrial mass of the injected CAR-T-cells was positively correlated with complete response, the absence of PD-1, TIM-3, and LAG-3, and the persistence of the T-cells [106]. This indicates that clinical outcome is linked to the mitochondrial mass of the CAR-T-cells and that therapeutic efficacy may be enhanced by promoting mitochondrial genesis during CAR-T-cell production [106].

### 3.4. Altered Amino Acid Metabolism Influences the Tumor Microenvironment

Amino acids also play a pivotal role in tumor development and progression as they are required for remodeling of stromal and vascular architecture in the inflamed microenvironment [111]. On one hand, the sensing of dying cells activates innate immune cells to produce interferons (IFNα, IFNβ, IFNγ, and TGFβ), which stimulate production of enzymes that catabolize tryptophan (Trp), such as Indoleamine 2,3 dioxygenase (IDO), and others that catabolize arginine (Arg), such as arginase 1 [111]. On the other hand, it was shown that malignant cells overexpress IDO induced by oncogenic RAS signaling, proinflammatory COX2 activation or BIN1 inactivation. High IDO expression on tumor cells correlates with poor outcome after therapy in several tumor types and with poor survival in acute myeloid leukemia [112,113,114,115,116,117].

The Arg catabolism product L-ornithine enables cells to generate polyamines, which might promote tumor development and force tumor-supporting immunity [118,119]. Products of Trp catabolism are known to be immune-suppressive, such as kynurenine (Kyn) and kynurenic acid, which are agonists of the aryl hydrogen receptor (AHR). AHR induces the expression of IDO on dendritic cells, induces generation of T_Reg_-cells, and supports tumor growth by regulation of oncogene expression, cell survival, and angiogenesis [120,121,122,123]. 3-hydroxyanthranilic acid, as another product of Trp catabolism, inhibits pyruvate dehydrogenase kinase (PDK1) and NF-κB activation, thereby blocking T-cell proliferation, promoting T-cell apoptosis, and activating T_Reg_-cells [124,125].

As an additional mechanism of increased T_Reg_ occurrence, CLL cells highly express CD200. Binding the CD200-receptor, CD200R, on T-cells, IDO is induced, leading to less active effector T-cells and T_Reg_ induction [126].

In total, malignant cells guide their own metabolism as well as immune cells and fibrocytes to catabolize more Trp and Arg, which results in immune-suppressive modulations of the tumor microenvironment. As a consequence of immune suppression, the clinical outcome of patients is significantly affected. Patients with non-small-cell-lung-cancer and low IDO activity treated with radiotherapy had a much longer survival than patients with high IDO activity [127]. Moreover, IDO inhibition or reduction of Kyn in the tumor microenvironment leads to a higher efficacy of cytotoxic drugs and immune checkpoint inhibitors in preclinical mouse models [128,129,130,131].

In Hodgkin lymphoma, histiocytes, macrophages, dendritic cells, and endothelial cells of the tumor microenvironment express IDO [132]. Higher IDO expression was found in EBV-positive cases, high Ann Arbor stages, high International Prognostic Score (IPS), and in patients having B-symptoms, while the mixed cellularity type of Hodgkin lymphoma showed the highest expression and the nodular sclerosis (NS) type predominantly showed low expression. Nevertheless, in both subtypes, a high IDO expression was associated with poor overall survival. In NS-type Hodgkin lymphoma, IDO expression could be seen as an independent prognostic factor. Patients with high IDO expression and frequent infiltration of CD163^+^ or CD68^+^ cells showed a five-year overall survival of just 67.8%, whereas patients with low IDO expression survived in 91.7% of cases [132].

**Figure 1:**
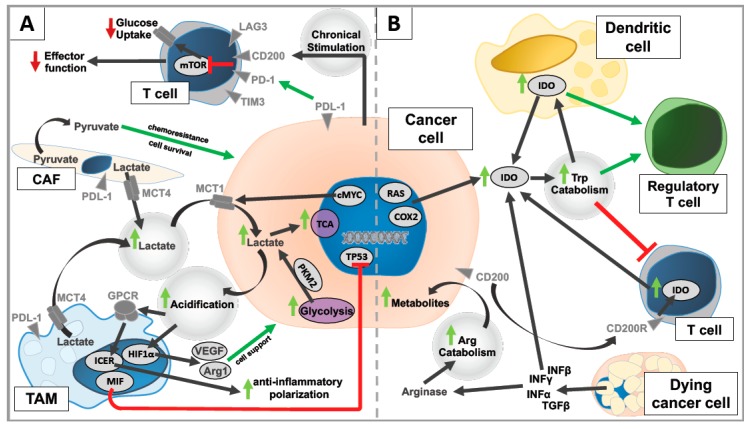
Graphic overview on the tumor microenvironment as a target of tumor metabolism (**A**) Influence of glycolytic alterations on the tumor microenvironment, (**B**) Influence of altered amino acid metabolism on the tumor microenvironment. Lime green arrows: Upregulation, Red arrows: Downregulation.

In conclusion, metabolites of altered pathways such as lactate as well as acidification and alterations of the amino acid metabolism exert a multitude of effects in nontransformed immune cells, mostly promoting tumor progression (Figure 1).

## 4. Therapeutic Approaches to Metabolism in B-Cell Malignancies

Specific alterations of malignant cells or tumor tissues frequently harbor the opportunity for therapeutic approaches. As such, tumor metabolism has similarly reached clinical care and numerous trials. Moreover, the tumor metabolism both in malignant B-cells as well as in the TME play a pivotal role for established therapeutic principles (Table 1).

### 4.1. Targeting Glycolysis

As acidification of the tumor microenvironment has that many negative effects on immune cells, diminishing the acidification as a therapeutic strategy is under investigation. Currently, a Phase I trial in diffuse large B-cell lymphoma and Burkitt lymphoma is utilizing the MCT1 inhibitor AZD3965, which inhibits lactate export into the tumor microenvironment and leads to lactate accumulation in tumor cells (NCT01791595). Moreover, a systemic buffering approach of acidification is under evaluation. In a murine B-cell lymphoma model, it was shown that NK-cell function increased and tumor growth was delayed after oral application of buffering bicarbonate [133,134]. Persistence of transferred T-cells could be improved by oral buffering in mice [135]. To overcome difficulties of systemic effects, new strategies are under investigation, such as calcium carbonate nanoparticles, which could be applied intravenously, that release bicarbonate in dependency of the local acidity [172].

Recent reports suggest the administration of mannose as having a positive impact on antitumor therapy. Mannose is taken up by the same transporters as glucose but cannot be degraded and accumulates as mannose-6-phosphate in the cell; subsequently glycolysis, the TCA cycle, the pentose-phosphate pathway, and glycan synthesis are inhibited [136,137]. Through this metabolic inhibition, proliferation of K562 leukemia and other tumor cell lines were decreased in vitro and in vivo. Mannose alone did not influence tumor cell viability, but it was shown that it significantly increases cell death and leads to significantly increased life expectancy in tumor-bearing nude mice when given in combination with cisplatin or doxorubicin [137]. This effect might be driven by a decrease of antiapoptotic proteins of the B-family after mannose application, leading to sensitization to cell death-inducing agents [137]. Further studies have to show if mannose application could also have a positive impact on the outcome of leukemia or lymphoma treatment.

A combined targeting of glycolysis and OxPhos is currently under investigation in several B-cell malignancies. The combined application of ritonavir, an inhibitor of the GLUT4 transporter and thereby glycolysis, in combination with metformin, inhibiting the electron transport chain (ETC) complex I and thereby OxPhos and leading to energy depletion thereby inhibiting the activation of mTORC1, leads to significant tumor regression in multiple myeloma mouse models and to cell death in primary CLL patient samples [138,139,140,173]. Under cotreatment, activation level of pAMPK, supporting oxidative phosphorylation, and of pAKT, supporting glycolysis, are decreased in multiple myeloma mouse [140,174,175]. Moreover, the phosphorylation of mTORC1 is suppressed, and lower pAKT and pmTORC1 levels lead to less MCL-1 expression [176]. High MCL-1 levels correlate with resistance to several chemotherapeutic agents and a lower event-free survival in multiple myeloma [140]. By using combinational therapy of ritonavir plus metformin in DLBCL and MCL cell lines, a decrease of cell growth was observed [140]. These results might indicate a second medical use of already FDA-approved drugs in clinical use for metabolism-directed therapies in B-cell malignancies. It has been shown that metformin increases the amount of tumor-infiltrating lymphocytes and the activity of CD8^+^ T-cells [141,142,143]. Moreover, the amount of T_Reg_-cells is diminished [144]. So far, it remains to be clarified if metformin has a tumor suppressive or supporting effect on TAMs or vascularization [177].

### 4.2. Targeting Isocitrate Dehydrogenase Mutation

In about 20% of the AML cases, a mutation in the isocitrate dehydrogenase (IDH) is found [16,178]. As described above, IDH mutation leads to 2-hydroxyglutarate (2-HG) production, and increased expression of HIF1α, thereby promoting DNA hypermethylation, resulting in impaired cellular differentiation and promotion of leukemia development and increased glycolysis for energy production [22,179]. To treat this mutation affecting cellular metabolism, several IDH inhibitors are currently under investigation. Clinical studies for newly diagnosed IDH1- or IDH2-mutated AML with IDH inhibitors used as monotherapy, in combination with intensive chemotherapy or with 5-azacitidine for unfit patients are running [21]. Enasidinib selectively inhibits IDH2, induces differentiation of leukemic cells, and leads to rapid improvement of absolute neutrophil count [15,145]. As enasidinib achieved high objective response rates, complete remission rates, and overall survival in refractory/relapsed AML with IDH2 mutation, FDA-approval was granted in 2017. Currently, a phase 3 clinical trial is evaluating enasidinib versus conventional care in older patients with IDH2-mutated AML and relapse or refractory disease after second- or third-line treatment (NCT02577406) [21]. As a selective inhibitor of IDH1, BAY1436032 is currently tested in a clinical phase 1 trial. BAY1436032 has shown suppression of 2-HG, induction of AML cell differentiation, inhibition of cell cycle progression and self-renewal of leukemic stem cells in vitro as in vivo in preclinical studies [146]. Furthermore, BAY1436032 is able to cross the blood–brain barrier [180]. As a dual inhibitor of IDH1 and IDH2, IDH-881 is being tested in a phase 1 clinical trial after showing reduction of 2-HG and the ability to cross the blood–brain barrier [21].

Due to the increase of 2-HG, IDH-mutated AML cells are highly dependent on antiapoptotic Bcl-2 [6]. 2-HG inhibits cytochrome-c oxidase, which would lead to activation of proapoptotic BAX and BAK and thereby to cell death, if Bcl-2 would not inhibit these proapoptotic factors [6].

### 4.3. Targeting PI3K Alterations

Regarding the dysregulated PI3K/AKT/mTORC1 pathway in B-cell malignancies, it was shown that the γ and δ isoforms of PI3K are overexpressed in the Hodgkin and Reed/Sternberg cells of Hodgkin lymphomas [147]. PI3Kδ is highly expressed in hematopoietic tissues and regulates survival, activation, proliferation, and homing of B-cells.

As an approved therapeutic agent, the PI3Kδ inhibitor idelalisib is approved for treatment of follicular lymphoma and CLL [148,149]. Regarding effects on the tumor microenvironment, on one hand, idelalisib increases the CD8^+^ T-cell response and inhibits T_reg_-cells; on the other hand, it decreases antibody-dependent phagocytosis by macrophages [150,151]. Currently, the dual PI3Kδ/γ inhibitor RP6530 is under clinical investigation in Phase II trials in Europe and the United States. RP6530 showed high antiproliferative and cytotoxic activity in Hodgkin lymphoma cell lines and xenograft mouse models [181]. The PI3Kδ/γ inhibitor downregulates genes involved in glycolysis, HIF1α-, MAPK-, JAK/STAT-, IL2-, IL4/STAT5-, MYC-signaling, and cell proliferation, wherein the most influencing downregulated gene is PKM2. Moreover, genes playing a role in cell death, apoptosis, and cell-cycle deregulation are upregulated [181]. In total, RP6530 leads to downregulated lactic acid production in lymphoma cells and in macrophages. By inhibition of lactate production in lymphoma cells, RP6530 interrupted immune-suppressive polarization of macrophages through cancer cells [181]. Immunosuppressive M2 macrophages were more sensitive to the PI3Kδ/γ inhibitor than proinflammatory M1 macrophages, most likely due to M2 macrophages harboring higher levels of PI3Kδ compared to M1 macrophages. By activating STAT1 phosphorylation and inhibiting STAT6 phosphorylation, RP6530 directly promotes proinflammatory M1 polarization [181]. Under dual PI3Kδ/γ inhibition, a decrease of macrophage-attracting chemokines (i.e., CSF-1, CCL5, TARC/CCL17) is seen and leads to a significant reduction of F4/80^+^ TAMs in Hodgkin lymphoma xenografts. The reduction of circulating suppressive monocytes in Hodgkin lymphoma patients’ blood under RP6530 treatment correlated with clinical outcome [181]. Responsive patients had significant reduction of thymus and activation-regulated cytokine (TARC), which is an M1 macrophage suppressor, so that TARC level might be used as a marker of response. The M1 induction through dual PI3Kδ/γ inhibition was also followed by strong reduction of proangiogenic factors (EGF, VEGF, HIF1α), leading to endothelial cell apoptosis, which might explain the increased necrotic areas in the tumors under RP6530 treatment [181].

In total, RP6530 reaches its therapeutic potential through direct cytostatic and cytotoxic effects on the lymphoma cells, a proinflammatory polarization of macrophages, depression of immuno-suppressive macrophages, and apoptosis of tumor-fueling blood vessels.

### 4.4. Targeting Fatty Acid Oxidation and Oxidative Phosphorylation

For treatment of CML and Ph^+^-B-ALL, BCR-ABL inhibitors, such as imatinib, are established treatment principles also utilizing metabolic downstream effects [152,153]. Through inhibition of the kinase domain, imatinib leads to strong suppression of glycolysis with decreased levels of HK2, LDHA, and PKM2, and lower expression of GLUT1, thereby leading to lower lactate secretion [154,155,182]. This might lead to a better immune response of the tumor microenvironment. Simultaneously, the CML cells upregulate OxPhos and fatty-acid oxidation (FAO), so that the ATP levels could be maintained [90,183]. Increase in FAO is achieved by upregulating the rate-limiting enzyme carnitine palmitoyl transferase 1C (CPT1C), and by activating the AMP-activated protein kinase (AMPK), which leads to acyl-CoA carboxylase (ACC) inhibition and thereby to FAO increase [184,185]. FAO not only rescues ATP levels, but also leads to chemoresistance [186]. These compensatory metabolic adaptations might lead to less efficacy of imatinib, a worse outcome, and the appearance of resistance or relapse.

Currently, AIC-47 is being tested in preclinical trials. AIC-47 not only inhibits BCR-ABL phosphorylation, but suppresses the expression of the protein itself [183]. AIC-47 also achieves cell death through inhibition of glycolysis. In contrast to imatinib, ATP levels are decreased by AIC-47. This might be due to the fact that after AIC-47 treatment, the expression of CPT1C and AMPK are decreased and thereby the compensatory upregulation of FAO is abrogated [183]. In a leukemia mouse model, AIC-47 led to higher decrease of hepatosplenomegaly than imatinib without significant toxicity [187]. As leukemia stem cells are maintained at least in part by FAO and are responsible for relapse, the use of AIC-47 might reveal superior results in comparison to imatinib [188]. Combining imatinib and AIC-47 treatment, strengthened inhibition of glycolysis, and synergistic cytotoxicity was observed in leukemia cell line K562 and KCL-22 and in human BCR/ABL-positive CML cells [183].

In total, AIC-47 might overcome imatinib resistance by preventing metabolic escape mechanisms, and might increase therapeutic success, used as single agent or in combination. However, this hypothesis remains to be elucidated in clinical trials.

In CLL, fatty acids are also used as a source for OxPhos [189]. STAT3 is constitutively activated in CLL cells and leads to lipoprotein lipase (LPL) expression, which increases the uptake of lipoproteins and their hydrolysis into free fatty acids (FFAs) [63,190,191]. The FFAs are used as fuel for OxPhos and moreover bind to PPAR-α, which thereby activates the transcription of enzymes necessary for OxPhos [62,192]. LPL expression is also promoted by BCR signaling and could be seen as a prognostic factor in CLL [156,193]. As a junction of several dysregulated signaling pathways in CLL, LPL might be a promising therapeutic target. Indeed, the lipase inhibitor orlistat induced apoptosis and cell death in primary CLL cells and showed additive cytotoxicity when combined with fludarabine [164].

In cases of ibrutinib resistance, a Bruton´s tyrosine kinase inhibitor approved for relapsed/refractory mantle cell lymphoma (MCL) and CLL, metabolic escape mechanisms have been identified.

Interestingly, upon ibrutinib resistance in MCL, no mutations in the BCR or NF-κB signaling pathway are found, but there is increased expression of genes involved in OxPhos, mTOR signaling, and cell cycle regulation and MYC-related genes [194]. A higher glutamine uptake, higher glutaminase expression, and a higher level of α-ketoglutarate could be seen in ibrutinib-resistant MCL cells, as well as a higher oxygen consumption rate [194]. In total, glutamine metabolism and OxPhos seem to be upregulated upon ibrutinib resistance and therefore might be potential therapeutic targets. Glutamine deprivation or inhibition of glutamine metabolism by amino-oxyacetate leads to a decreased oxygen consumption rate and the induction of ROS and energy stress in the resistant cells, suggesting that glutamine is the primary substrate for OxPhos and antioxidative glutathione production is needed in resistant MCL cells to maintain redox balance upon increased mitochondrial activity [194]. Inhibition of the ETC complex I by IACS-010759 decreases proliferation in the resistant MCL cells. By combining ETC complex I inhibition and glutamine metabolism inhibition, even greater ROS production could be observed.

In summary, increased mitochondrial OxPhos energy production might be the metabolic escape mechanism in ibrutinib-resistant MCL cells [194]. This mechanism could be driven by the upregulation of the glutamine transporter SLC1A5 in resistant cells, which leads to increased glutamine uptake and the activation of mTORC1 signaling [194,195]. mTORC1 itself regulates glutamine metabolism via MYC and glutaminase. This results in increased glutamine uptake; as such, ibrutinib-resistant MCL cells might induce a potential feedback-mechanism to escape from BTK-signaling reliance for energy production [56]. In an ibrutinib-resistant MCL PDX mouse model generated out of primary ibrutinib-resistant MCL cells, inhibition of ETC complex I by IACS-010759 led to complete inhibition of tumor growth and extended survival without any apparent toxicities [194]. This result was also achieved by using a PDX mouse model created from ibrutinib-resistant primary cells of a double-hit (MYC, BCL-2) B-cell lymphoma [194]. Currently, a Phase I trial with IACS-010759 is ongoing.

In the case of ibrutinib resistance in CLL, it was observed that mutations most frequently occur in BTK or its target PLC2 [165]. Nevertheless, a decrease in glutamate secretion was observed in resistant cells upon ibrutinib treatment [196]. Moreover, an increase of FAO in CLL cells is described and an inhibition of free fatty acid synthesis through ibrutinib has been reported [166,189,197]. Through glutamate accumulation fueling the TCA cycle and increasing FAO, resistant CLL cells might preserve their energy production. To inhibit FAO and overcome resistance, the carnitine palmitoyl transferase (CPT1) inhibitor etomoxir was evaluated and achieved resensitization towards ibrutinib-induced cytotoxicity in ibrutinib-resistant CLL patient samples [196]. Moreover, etomoxir leads to suppression of immune-suppressive polarization of macrophages; thereby, antitumor immunity might be increased [198]. Unfortunately, etomoxir led to severe hepatotoxicity in clinical practice, but the inhibition of FAO might still be a promising concept.

Inhibition of the glutamine metabolism might also be a target in ibrutinib-sensitive CLL cells, as an increased sensitivity to glutaminase inhibition was observed in del11q-positive cells [157].

Ibrutinib itself has a wide influence also on the tumor microenvironment. It leads to less CD200 and BTLA expression in CLL cells and to less IL-10 secretion; therefore, immunosuppression might be decreased [158]. After ibrutinib treatment, an increase of CD4^+^ and CD8^+^ T-cells, particularly along with tumor antigen-specific T-cells, is observed in CLL patient samples. This might be mediated through the decrease of activation-induced cell death (AICD) upon ibrutinib application [158]. Moreover, ibrutinib leads to increased response of CD8^+^ T-cells in vitro and in vivo through inhibition of IL2-inducible T-cell kinase (ITK), and increases Th17 cells, leading to increased dendritic cell response and improved survival in CLL patients [159,160,199]. CD4^+^ and CD8^+^ T-cells express less PD-1 and CTL-4 upon ibrutinib treatment and in the context of lymphoma mouse models, an increased T-cell antitumor immune response as well as potentiated efficacy of immune checkpoint blockade could be observed after ibrutinib application [158,200,201]. Here, it is noteworthy that an increased response of anti-CD19 CAR-T-cell therapy was observed in MCL when ibrutinib was added [161]. As another branch of potential antitumor effector immune cells, NK-cells were also rescued from AICD by ibrutinib. Macrophages were polarized toward CD8^+^ T-cell supporting and away from an immune-suppressive phenotype but their phagocytosis rates have been described to be decreased [151,158,162,163,202,203]. Of note, most of these effects on the tumor microenvironment could not be achieved with the second generation BTK inhibitor, acalabrutinib, which is more selective to BTK and, in contrast to ibrutinib, does not target ITK [158].

### 4.5. Targeting the Pentose-Phosphate Pathway

In multiple myeloma (MM), resistance to melphalan can appear due to metabolic changes. In the resistant cells, most of the glycolytic and pentose-phosphate pathway (PPP) enzymes are upregulated, whereas proteins of the TCA cycle and ETC are downregulated [204]. This switch is also seen in cancer cells using the Warburg effect [167]. The PPP-enzyme glucose-6-phosphate-dehydrogenase is the most upregulated enzyme in melphalan-resistant MM cell lines. The pyruvate dehydrogenase kinase inhibitor dichloracetate, used for glycolysis inhibition, leads to increased ROS in the resistant cells, and, in combination with melphalan, to induction of apoptosis [204]. In the resistant cells, a higher level of lactate but less lactate secretion was observed. Higher lactate levels might be used to produce a pseudohypoxic condition, which leads to upregulation of NF-κB/IL-8 and VEGF signaling [204]. Higher IL-8 and VEGF was observed in melphalan-resistant MM cell lines and this might lead to promotion of survival and proliferation by induction of PI3K/mTOR and STAT3 signaling. Indeed, PI3K inhibition led to cytotoxicity in the resistant cells, underlying increased IL-8/VEGF-signaling and subsequent upregulation in glycolytic pathways as a possible resistance mechanism [204].

### 4.6. Targeting Amino Acid Metabolism

Targeting amino acid metabolism is also under investigation as a strategy of treatment in AML. AML stem cells rely on oxidative phosphorylation for survival [169]. Stem cells utilize cysteine for glutathione synthesis, which is then required for glutathionylation of succinate dehydrogenase A (SDHA). SDHA serves as a component of the electron transport chain. Upon cysteine depletion, ATP production particularly in AML stem cells is impaired, thereby leading to cell death [168]. Recently, venetoclax + azacitidine was approved for first line therapy in AML. It was shown that this regime decreased levels of cysteine and glutathione, as well as the glutathionylation of SDHA in the leukemic cells [169,205]. Upon resistance to venetoclax + azacitidine, no more decrease of the metabolites is seen, indicating that the stem cells might employ this metabolic pathway as an escape strategy [168,169,205]. This resistance mechanism may be countered by attribution of cyst(e)inase, a cysteine degrading enzyme, which also eradicates stem cells in AML patient samples without having negative effects on normal human stem cells [168].

In venetoclax resistance of CLL, genomic alteration of PD-L1 has been detected but the respective functional impact remains to be elucidated [206].

Finally, ALL relies on supply of amino acids—particularly asparagine for cell growth and survival—while ALL cells express asparagine synthetase at a low level [170]. In this line, the use of pegaspargase is an established clinical practice, which hydrolyses asparagine into aspartic acid and ammonia and is implemented in a multiagent chemotherapy in first-line treatment of ALL [171,207]. Here, pegaspargase was shown to mediate inhibited protein, DNA-, and RNA-synthesis, and to induce cell death in ALL [208]

## 5. Metabolism in B-Cell Malignancy—Exploiting Specific Vulnerabilities for Tailored Therapy

In conclusion, it has become obvious that metabolic processes are a distinct hallmark of cancer. The functional impact of altered tumor metabolism is not only limited to the compartment of malignant cells but has to be assessed with the perspective of the tumor microenvironment and immune regulation. Summarizing the various modes of influencing metabolism in B-cell malignancies, alterations of oxidative phosphorylation, glucose, and fatty acid metabolism stand out. With these results at hand, translational approaches have been implemented with promising results and first clinical implementations. Our deepened understanding of tumor metabolism has also connected novel findings in the tumor metabolism field to already established therapeutic concepts. Targeting metabolism to influence the cancer cells and the tumor microenvironment has already become an established therapeutic principle in hematological cancer treatment and might bear large potential for new effective and better tolerated therapeutic strategies in newly diagnosed and relapsed/refractory B-cell malignancies. Improving the metabolomic methodology and functional assessment of metabolism in B-cell malignancy remains a promising challenge in the field towards improved diagnostics and therapy.

## Figures and Tables

**Table 1:** Therapeutical agents and their effects on tumor cells and the TME in B-cell malignancies.

Substance	Mechanism	Phase	Effect on Tumor Cells	Effect on Microenvironment	Reference
AZD3965	MCT1/MCT2 inhibition	Phase I trial currently running	proliferation inhibition	decreased acidification, increased immune response by macrophages and T-cells	NCT01791595
bicarbonate	countering acidification	preclinical	proliferation inhibition	increased NK-cell function, longer persistence of transferred T-cells	[133,134,135]
mannose	inhibition of glycolysis, TCA, PPP, glycan synthesis; inhibition of anti-apoptotic proteins	preclinical	proliferation inhibition, cell death sensitization	(not known)	[136,137]
ritonavir + metformin	GLUT4 inhibition + ETC complex I inhibition	Approved for nonmalignant indication	CLL cell death, MM tumor growth arrest, DLBCL cell line growth arrest, MCL cell line growth arrest	decreased PD-L1 expression, decreased T_reg_ infiltration, increased CD8^+^ T-cell function	[138,139,140,141,142,143,144]
enasidinib	IDH2 inhibition	approved for RR-AML	AML cell differentiation	improved neutrophil count	[15,145]
BAY1436032	IDH1 inhibition	Phase I trial currently running	AML cell differentiation, inhibition of AML stem cell proliferation	(not known)	[146]
IDH-881	IDH1 + IDH2 inhibition	Phase I trial currently running	AML cell differentiation	(not known)	[21]
Idelalisib	PI3Kδ inhibition	approved for FL and CLL	cell death	Treg inhibition, increased CD8^+^ T-cell response, decreased antibody-dependent phagocytosis	[147,148,149,150]
RP6530	PI3Kδ and -γ inhibition	Phase II trial currently running	cell death	M1 polarization, M2 suppression, blood vessel apoptosis	[151]
Imatinib	inhibition of glycolysis	approved for BCR/ABL^+^-CML and B-ALL	cell death	decreased acidification	[152,153,154]
AIC-47	inhibition of glycolysis, inhibition of FAO	preclinical	cell death	decreased acidification	[155]
Orlistat	lipase inhibition	Approved for nonmalignant indication against obesity	CLL cell death	(not known)	[156]
Ibrutinib	Bruton´s tyrosine kinase inhibition	approved for CLL and RR-MCL	proliferation inhibition, decreased CD200 and BTLA expression	increased CD4+, CD8+ and Th17 cells, increased dendritic cell response, decreased PD-1, CTL4 and IL-10 expression, NK-cell rescue, decreased phagocytosis of macrophages and NK-cells	[150,157,158,159,160,161,162,163]
IACS-010759	ETC I inhibition	Phase I trial currently running	cell growth arrest	(not known)	[164]
Etomoxir	CPT-1 inhibition	no more in clinical use	resensitization to Ibrutinib in CLL cells	Inhibition of M2 polarization	[165,166]
venetoclax + azacitidine	inhibition of glutathione synthesis	approved for AML	stem cell death	(not known)	[167,168]
cyst(e)inase	inhibition of glutathione synthesis	preclinical	stem cell death (also in venetoclax + azacitidine resistant cells)	(not known)	[169]
Pegaspargase	asparagine degradation	approved for ALL	cell death	(not known)	[170,171]

## References

[1] Hanahan D., Weinberg R.A. (2000). The hallmarks of cancer. Cell.

[2] Hanahan D., Weinberg R.A. (2011). Hallmarks of cancer: the next generation. Cell.

[3] Pavlova N.N., Thompson C.B. (2016). The Emerging Hallmarks of Cancer Metabolism. Cell Metab..

[4] Warburg O. (1924). Über den Stoffwechsel der Carcinomzelle. Naturwissenschaften.

[5] M V.H., L C., C T. (2009). Understanding the Warburg Effect: the metabolic requirements of cell proliferation. Science.

[6] Corces-Zimmerman M.R., Xavy S., Zhao F., Majeti R., Chan S.M., Rastogi S., Medeiros B.C., Hong W.-J., Thomas D., Tyvoll D.A. (2015). Isocitrate dehydrogenase 1 and 2 mutations induce BCL-2 dependence in acute myeloid leukemia. Nat. Med..

[7] Dang L., White D.W., Gross S., Bennett B.D., Bittinger M.A., Driggers E.M., Fantin V.R., Jang H.G., Jin S., Keenan M.C. (2009). Cancer-associated IDH1 mutations produce 2-hydroxyglutarate. Nature.

[8] Kobe C., Kuhnert G., Kahraman D., Haverkamp H., Eich H.T., Franke M., Persigehl T., Klutmann S., Amthauer H., Bockisch A. (2014). Assessment of tumor size reduction improves outcome prediction of positron emission tomography/computed tomography after chemotherapy in advanced-stage Hodgkin lymphoma. J. Clin. Oncol..

[9] Pallasch C.P., Leskov I., Braun C.J., Vorholt D., Drake A., Soto-Feliciano Y.M., Bent E.H., Schwamb J., Iliopoulou B., Kutsch N. (2014). Sensitizing protective tumor microenvironments to antibody-mediated therapy. Cell.

[10] Hude I., Sasse S., Engert A., Bröckelmann P.J. (2017). The emerging role of immune checkpoint inhibition in malignant lymphoma. Haematologica.

[11] Bröckelmann P.J., Engert A. (2017). Checkpoint Inhibition in Hodgkin Lymphoma—A Review. Oncol. Res. Treat..

[12] Brahmer J., Reckamp K.L., Baas P., Crinò L., Eberhardt W.E.E., Poddubskaya E., Antonia S., Pluzanski A., Vokes E.E., Holgado E. (2015). Nivolumab versus Docetaxel in Advanced Squamous-Cell Non–Small-Cell Lung Cancer. N. Engl. J. Med..

[13] Weber J., Mandala M., Del Vecchio M., Gogas H.J., Arance A.M., Cowey C.L., Dalle S., Schenker M., Chiarion-Sileni V., Marquez-Rodas I. (2017). Adjuvant Nivolumab versus Ipilimumab in Resected Stage III or IV Melanoma. N. Engl. J. Med..

[14] Lossos C., Liu Y., Kolb K.E., Christie A.L., van Scoyk A., Prakadan S.M., Shigemori K., Stevenson K.E., Morrow S., Plana O.D. (2019). Mechanisms of lymphoma clearance induced by high-dose alkylating agents. Cancer Discov..

[15] Stein E.M., DiNardo C.D., Pollyea D.A., Fathi A.T., Roboz G.J., Altman J.K., Stone R.M., DeAngelo D.J., Levine R.L., Flinn I.W. (2017). Enasidenib in mutant *IDH2* relapsed or refractory acute myeloid leukemia. Blood.

[16] Mardis E.R., Ding L., Dooling D.J., Larson D.E., McLellan M.D., Chen K., Koboldt D.C., Fulton R.S., Delehaunty K.D., McGrath S.D. (2009). Recurring Mutations Found by Sequencing an Acute Myeloid Leukemia Genome. N. Engl. J. Med..

[17] Corces-Zimmerman M.R., Hong W.-J., Weissman I.L., Medeiros B.C., Majeti R. (2014). Preleukemic mutations in human acute myeloid leukemia affect epigenetic regulators and persist in remission. Proc. Natl. Acad. Sci. USA.

[18] Reitman Z.J., Yan H. (2010). Isocitrate dehydrogenase 1 and 2 mutations in cancer: alterations at a crossroads of cellular metabolism. J. Natl. Cancer Inst..

[19] Gorrini C., Harris I.S., Mak T.W. (2013). Modulation of oxidative stress as an anticancer strategy. Nat. Rev. Drug Discov..

[20] Lee S.M., Koh H.-J., Park D.-C., Song B.J., Huh T.-L., Park J.-W. (2002). Cytosolic NADP(+)-dependent isocitrate dehydrogenase status modulates oxidative damage to cells. Free Radic. Biol. Med..

[21] Nassereddine S., Lap C.J., Haroun F., Tabbara I. (2017). The role of mutant IDH1 and IDH2 inhibitors in the treatment of acute myeloid leukemia. Ann. Hematol..

[22] Chaturvedi A., Araujo Cruz M.M., Jyotsana N., Sharma A., Goparaju R., Schwarzer A., Görlich K., Schottmann R., Struys E.A., Jansen E.E. (2016). Enantiomer-specific and paracrine leukemogenicity of mutant IDH metabolite 2-hydroxyglutarate. Leukemia.

[23] Grassian A.R., Parker S.J., Davidson S.M., Divakaruni A.S., Green C.R., Zhang X., Slocum K.L., Pu M., Lin F., Vickers C. (2014). IDH1 mutations alter citric acid cycle metabolism and increase dependence on oxidative mitochondrial metabolism. Cancer Res..

[24] Oizel K., Gratas C., Nadaradjane A., Oliver L., Vallette F.M., Pecqueur C. (2015). D-2-Hydroxyglutarate does not mimic all the IDH mutation effects, in particular the reduced etoposide-triggered apoptosis mediated by an alteration in mitochondrial NADH. Cell Death Dis..

[25] Carmeliet P., Dor Y., Herbert J.M., Fukumura D., Brusselmans K., Dewerchin M., Neeman M., Bono F., Abramovitch R., Maxwell P. (1998). Role of HIF-1alpha in hypoxia-mediated apoptosis, cell proliferation and tumour angiogenesis. Nature.

[26] Chapuy B., Stewart C., Dunford A.J., Kim J., Kamburov A., Redd R.A., Lawrence M.S., Roemer M.G.M., Li A.J., Ziepert M. (2018). Molecular subtypes of diffuse large B cell lymphoma are associated with distinct pathogenic mechanisms and outcomes. Nat. Med..

[27] Landau D.A., Tausch E., Taylor-Weiner A.N., Stewart C., Reiter J.G., Bahlo J., Kluth S., Bozic I., Lawrence M., Böttcher S. (2015). Mutations driving CLL and their evolution in progression and relapse. Nature.

[28] Hamlyn P.H., Rabbitts T.H. (1983). Translocation joins c-myc and immunoglobulin γ1 genes in a Burkitt lymphoma revealing a third exon in the c-myc oncogene. Nature.

[29] Dejure F.R., Eilers M. (2017). MYC and tumor metabolism: chicken and egg. EMBO J..

[30] Liu Y.C., Li F., Handler J., Huang C.R.L., Xiang Y., Neretti N., Sedivy J.M., Zeller K.I., Dang C.V. (2008). Global regulation of nucleotide biosynthetic genes by c-myc. PLoS One.

[31] Morrish F., Neretti N., Sedivy J.M., Hockenbery D.M. (2008). The oncogene c-Myc coordinates regulation of metabolic networks to enable rapid cell cycle entry. Cell Cycle.

[32] Dejure F.R., Royla N., Herold S., Kalb J., Walz S., Ade C.P., Mastrobuoni G., Vanselow J.T., Schlosser A., Wolf E. (2017). The MYC mRNA 3′-UTR couples RNA polymerase II function to glutamine and ribonucleotide levels. EMBO J..

[33] Gao P., Tchernyshyov I., Chang T.C., Lee Y.S., Kita K., Ochi T., Zeller K.I., De Marzo A.M., Van Eyk J.E., Mendell J.T. (2009). C-Myc suppression of miR-23a/b enhances mitochondrial glutaminase expression and glutamine metabolism. Nature.

[34] Osthus R.C., Shim H., Kim S., Li Q., Reddy R., Mukherjee M., Xu Y., Wonsey D., Lee L.A., Dang C.V. (2000). Deregulation of glucose transporter 1 and glycolytic gene expression by c-Myc. J. Biol. Chem..

[35] Eberlin L.S., Gabay M., Fan A.C., Gouw A.M., Tibshirani R.J., Felsher D.W., Zare R.N. (2014). Alteration of the lipid profile in lymphomas induced by MYC overexpression. Proc. Natl. Acad. Sci. USA.

[36] Broecker-Preuss M., Becher-Boveleth N., Bockisch A., Duhrsen U., Muller S. (2017). Regulation of glucose uptake in lymphoma cell lines by c-MYC- and PI3K-dependent signaling pathways and impact of glycolytic pathways on cell viability. J. Transl. Med..

[37] Nilsson J.A., Keller U.B., Baudino T.A., Yang C., Norton S., Old J.A., Nilsson L.M., Neale G., Kramer D.L., Porter C.W. (2005). Targeting ornithine decarboxylase in Myc-induced lymphomagenesis prevents tumor formation. Cancer Cell.

[38] Le A., Lane A.N., Hamaker M., Bose S., Gouw A., Barbi J., Tsukamoto T., Rojas C.J., Slusher B.S., Zhang H. (2012). Glucose-independent glutamine metabolism via TCA cycling for proliferation and survival in B cells. Cell Metab..

[39] Doughty C.A., Bleiman B.F., Wagner D.J., Dufort F.J., Mataraza J.M., Roberts M.F., Chiles T.C. (2006). Antigen receptor-mediated changes in glucose metabolism in B lymphocytes: role of phosphatidylinositol 3-kinase signaling in the glycolytic control of growth. Blood.

[40] Vangapandu H.V., Havranek O., Ayres M.L., Kaipparettu B.A., Balakrishnan K., Wierda W.G., Keating M.J., Davis R.E., Stellrecht C.M., Gandhi V. (2017). B-cell Receptor Signaling Regulates Metabolism in Chronic Lymphocytic Leukemia. Mol. Cancer Res..

[41] Engelman J.A., Luo J., Cantley L.C. (2006). The evolution of phosphatidylinositol 3-kinases as regulators of growth and metabolism. Nat. Rev. Genet..

[42] Hahn-Windgassen A., Nogueira V., Chen C.-C., Skeen J.E., Sonenberg N., Hay N. (2005). Akt activates the mammalian target of rapamycin by regulating cellular ATP level and AMPK activity. J. Biol. Chem..

[43] Robey R.B., Hay N. (2009). Is Akt the “Warburg kinase”?-Akt-energy metabolism interactions and oncogenesis. Semin. Cancer Biol..

[44] Ricci J.-E., Chiche J. (2018). Metabolic Reprogramming of Non-Hodgkin’s B-Cell Lymphomas and Potential Therapeutic Strategies. Front. Oncol..

[45] Wang X., Cao X., Sun R., Tang C., Tzankov A., Zhang J., Manyam G.C., Xiao M., Miao Y., Jabbar K. (2018). Clinical Significance of PTEN Deletion, Mutation, and Loss of PTEN Expression in De Novo Diffuse Large B-Cell Lymphoma. Neoplasia.

[46] Coiffier B., Ribrag V. (2009). Exploring mammalian target of rapamycin (mTOR) inhibition for treatment of mantle cell lymphoma and other hematologic malignancies. Leuk. Lymphoma.

[47] Ma Y., Zhang P., Gao Y., Fan H., Zhang M., Wu J. (2015). Evaluation of AKT phosphorylation and PTEN loss and their correlation with the resistance of rituximab in DLBCL. Int. J. Clin. Exp. Pathol..

[48] Okosun J., Wolfson R.L., Wang J., Araf S., Wilkins L., Castellano B.M., Escudero-Ibarz L., Al Seraihi A.F., Richter J., Bernhart S.H. (2016). Recurrent mTORC1-activating RRAGC mutations in follicular lymphoma. Nat. Genet..

[49] Jewell J.L., Kim Y.C., Russell R.C., Yu F.-X., Park H.W., Plouffe S.W., Tagliabracci V.S., Guan K.-L. (2015). Differential regulation of mTORC1 by leucine and glutamine. Science.

[50] Ma X.M., Blenis J. (2009). Molecular mechanisms of mTOR-mediated translational control. Nat. Rev. Mol. Cell Biol..

[51] Ben-Sahra I., Howell J.J., Asara J.M., Manning B.D. (2013). Stimulation of de novo pyrimidine synthesis by growth signaling through mTOR and S6K1. Science.

[52] Düvel K., Yecies J.L., Menon S., Raman P., Lipovsky A.I., Souza A.L., Triantafellow E., Ma Q., Gorski R., Cleaver S. (2010). Activation of a Metabolic Gene Regulatory Network Downstream of mTOR Complex 1. Mol. Cell.

[53] Cunningham J.T., Rodgers J.T., Arlow D.H., Vazquez F., Mootha V.K., Puigserver P. (2007). mTOR controls mitochondrial oxidative function through a YY1–PGC-1α transcriptional complex. Nature.

[54] Morita M., Gravel S.-P., Chénard V., Sikström K., Zheng L., Alain T., Gandin V., Avizonis D., Arguello M., Zakaria C. (2013). mTORC1 Controls Mitochondrial Activity and Biogenesis through 4E-BP-Dependent Translational Regulation. Cell Metab..

[55] Csibi A., Fendt S.-M., Li C., Poulogiannis G., Choo A.Y., Chapski D.J., Jeong S.M., Dempsey J.M., Parkhitko A., Morrison T. (2013). The mTORC1 Pathway Stimulates Glutamine Metabolism and Cell Proliferation by Repressing SIRT4. Cell.

[56] Csibi A., Lee G., Yoon S.-O., Tong H., Ilter D., Elia I., Fendt S.-M., Roberts T.M., Blenis J. (2014). The mTORC1/S6K1 pathway regulates glutamine metabolism through the eIF4B-dependent control of c-Myc translation. Curr. Biol..

[57] Lewis B.C., Prescott J.E., Campbell S.E., Shim H., Orlowski R.Z., Dang C. (2000). V Tumor induction by the c-Myc target genes rcl and lactate dehydrogenase A. Cancer Res..

[58] Denko N.C. (2008). Hypoxia, HIF1 and glucose metabolism in the solid tumour. Nat. Rev. Cancer.

[59] Perez-Galan P., Dreyling M., Wiestner A. (2011). Mantle cell lymphoma: biology, pathogenesis, and the molecular basis of treatment in the genomic era. Blood.

[60] Reddy A., Zhang J., Davis N.S., Moffitt A.B., Love C.L., Waldrop A., Leppa S., Pasanen A., Meriranta L., Karjalainen-Lindsberg M.-L. (2017). Genetic and Functional Drivers of Diffuse Large B Cell Lymphoma. Cell.

[61] Xiao G., Chan L.N., Klemm L., Braas D., Chen Z., Geng H., Zhang Q.C., Aghajanirefah A., Cosgun K.N., Sadras T. (2018). B-Cell-Specific Diversion of Glucose Carbon Utilization Reveals a Unique Vulnerability in B Cell Malignancies. Cell.

[62] Rozovski U., Hazan-Halevy I., Barzilai M., Keating M.J., Estrov Z. (2016). Metabolism pathways in chronic lymphocytic leukemia. Leuk. Lymphoma.

[63] Rozovski U., Grgurevic S., Bueso-Ramos C., Harris D.M., Li P., Liu Z., Wu J.Y., Jain P., Wierda W., Burger J. (2015). Aberrant LPL Expression, Driven by STAT3, Mediates Free Fatty Acid Metabolism in CLL Cells. Mol. Cancer Res..

[64] Wheeler M.L., Defranco A.L. (2012). Prolonged production of reactive oxygen species in response to B cell receptor stimulation promotes B cell activation and proliferation. J. Immunol..

[65] Capasso M., Bhamrah M.K., Henley T., Boyd R.S., Langlais C., Cain K., Dinsdale D., Pulford K., Khan M., Musset B. (2010). HVCN1 modulates BCR signal strength via regulation of BCR-dependent generation of reactive oxygen species. Nat. Immunol..

[66] Falchi L., Keating M.J., Marom E.M., Truong M.T., Schlette E.J., Sargent R.L., Trinh L., Wang X., Smith S.C., Jain N. (2014). Correlation between FDG/PET, histology, characteristics, and survival in 332 patients with chronic lymphoid leukemia. Blood.

[67] Hartmann S., Agostinelli C., Diener J., Doring C., Fanti S., Zinzani P.L., Gallamini A., Bergmann L., Pileri S., Hansmann M.L. (2012). GLUT1 expression patterns in different Hodgkin lymphoma subtypes and progressively transformed germinal centers. BMC Cancer.

[68] Christofk H.R., Vander Heiden M.G., Harris M.H., Ramanathan A., Gerszten R.E., Wei R., Fleming M.D., Schreiber S.L., Cantley L.C. (2008). The M2 splice isoform of pyruvate kinase is important for cancer metabolism and tumour growth. Nature.

[69] Colegio O.R., Chu N.Q., Szabo A.L., Chu T., Rhebergen A.M., Jairam V., Cyrus N., Brokowski C.E., Eisenbarth S.C., Phillips G.M. (2014). Functional polarization of tumour-associated macrophages by tumour-derived lactic acid. Nature.

[70] Bohn T., Rapp S., Luther N., Klein M., Bruehl T.J., Kojima N., Aranda Lopez P., Hahlbrock J., Muth S., Endo S. (2018). Tumor immunoevasion via acidosis-dependent induction of regulatory tumor-associated macrophages. Nat. Immunol..

[71] Harzenetter M.D., Novotny A.R., Gais P., Molina C.A., Altmayr F., Holzmann B. (2007). Negative regulation of TLR responses by the neuropeptide CGRP is mediated by the transcriptional repressor ICER. J. Immunol..

[72] Porta C., Rimoldi M., Raes G., Brys L., Ghezzi P., Di Liberto D., Dieli F., Ghisletti S., Natoli G., De Baetselier P. (2009). Tolerance and M2 (alternative) macrophage polarization are related processes orchestrated by p50 nuclear factor B. Proc. Natl. Acad. Sci. USA.

[73] Chang C.I., Liao J.C., Kuo L. (2001). Macrophage arginase promotes tumor cell growth and suppresses nitric oxide-mediated tumor cytotoxicity. Cancer Res..

[74] Noman M.Z., Desantis G., Janji B., Hasmim M., Karray S., Dessen P., Bronte V., Chouaib S. (2014). PD-L1 is a novel direct target of HIF-1α, and its blockade under hypoxia enhanced MDSC-mediated T cell activation. J. Exp. Med..

[75] Liu D., Chang C., Lu N., Wang X., Lu Q., Ren X., Ren P., Zhao D., Wang L., Zhu Y. (2017). Comprehensive Proteomics Analysis Reveals Metabolic Reprogramming of Tumor-Associated Macrophages Stimulated by the Tumor Microenvironment. J. Proteome Res..

[76] Hudson J.D., Shoaibi M.A., Maestro R., Carnero A., Hannon G.J., Beach D.H. (1999). A Proinflammatory Cytokine Inhibits p53 Tumor Suppressor Activity. J. Exp. Med..

[77] Weide B., Martens A., Hassel J.C., Berking C., Postow M.A., Bisschop K., Simeone E., Mangana J., Schilling B., Di Giacomo A.M. (2016). Baseline Biomarkers for Outcome of Melanoma Patients Treated with Pembrolizumab. Clin. Cancer Res..

[78] Petrelli F., Cabiddu M., Coinu A., Borgonovo K., Ghilardi M., Lonati V., Barni S. (2015). Prognostic role of lactate dehydrogenase in solid tumors: A systematic review and meta-analysis of 76 studies. Acta Oncol..

[79] Diem S., Kasenda B., Spain L., Martin-Liberal J., Marconcini R., Gore M., Larkin J. (2016). Serum lactate dehydrogenase as an early marker for outcome in patients treated with anti-PD-1 therapy in metastatic melanoma. Br. J. Cancer.

[80] Sakamoto A., Kunou S., Shimada K., Tsunoda M., Aoki T., Iriyama C., Tomita A., Nakamura S., Hayakawa F., Kiyoi H. (2019). Pyruvate secreted from patient-derived cancer-associated fibroblasts supports survival of primary lymphoma cells. Cancer Sci..

[81] Aoki T., Shimada K., Sakamoto A., Sugimoto K., Morishita T., Kojima Y., Shimada S., Kato S., Iriyama C., Kuno S. (2017). Emetine elicits apoptosis of intractable B-cell lymphoma cells with MYC rearrangement through inhibition of glycolytic metabolism. Oncotarget.

[82] Afonso J., Pinto T., Simões-Sousa S., Schmitt F., Longatto-Filho A., Pinheiro C., Marques H., Baltazar F. (2019). Clinical significance of metabolism-related biomarkers in non-Hodgkin lymphoma—MCT1 as potential target in diffuse large B cell lymphoma. Cell. Oncol..

[83] Doherty J.R., Yang C., Scott K.E.N., Cameron M.D., Fallahi M., Li W., Hall M.A., Amelio A.L., Mishra J.K., Li F. (2014). Blocking lactate export by inhibiting the Myc target MCT1 Disables glycolysis and glutathione synthesis. Cancer Res..

[84] Gan L., Xiu R., Ren P., Yue M., Su H., Guo G., Xiao D., Yu J., Jiang H., Liu H. (2016). Metabolic targeting of oncogene MYC by selective activation of the proton-coupled monocarboxylate family of transporters. Oncogene.

[85] Martinez-Outschoorn U.E., Lisanti M.P., Sotgia F. (2014). Catabolic cancer-associated fibroblasts transfer energy and biomass to anabolic cancer cells, fueling tumor growth. Semin. Cancer Biol..

[86] Mikkilineni L., Whitaker-Menezes D., Domingo-Vidal M., Sprandio J., Avena P., Cotzia P., Dulau-Florea A., Gong J., Uppal G., Zhan T. (2017). Hodgkin lymphoma: A complex metabolic ecosystem with glycolytic reprogramming of the tumor microenvironment. Semin. Oncol..

[87] Halestrap A.P. (2013). The SLC16 gene family—Structure, role and regulation in health and disease. Mol. Aspects Med..

[88] Xin X., Zeng X., Gu H., Li M., Tan H., Jin Z., Hua T., Shi R., Wang H. (2016). CD147/EMMPRIN overexpression and prognosis in cancer: A systematic review and meta-analysis. Sci. Rep..

[89] Gao H., Jiang Q., Han Y., Peng J., Wang C. (2015). shRNA-mediated EMMPRIN silencing inhibits human leukemic monocyte lymphoma U937 cell proliferation and increases chemosensitivity to adriamycin. Cell Biochem. Biophys..

[90] Schmidt J., Bonzheim I., Steinhilber J., Montes-Mojarro I.A., Ortiz-Hidalgo C., Klapper W., Fend F., Quintanilla-Martínez L. (2017). EMMPRIN (CD147) is induced by C/EBPβ and is differentially expressed in ALK+ and ALK− anaplastic large-cell lymphoma. Lab. Investig..

[91] Siska P.J., van der Windt G.J.W., Kishton R.J., Cohen S., Eisner W., MacIver N.J., Kater A.P., Weinberg J.B., Rathmell J.C. (2016). Suppression of Glut1 and Glucose Metabolism by Decreased Akt/mTORC1 Signaling Drives T Cell Impairment in B Cell Leukemia. J. Immunol..

[92] MacIver N.J., Michalek R.D., Rathmell J.C. (2013). Metabolic Regulation of T Lymphocytes. Annu. Rev. Immunol..

[93] Jacobs S.R., Herman C.E., MacIver N.J., Wofford J.A., Wieman H.L., Hammen J.J., Rathmell J.C. (2008). Glucose Uptake Is Limiting in T Cell Activation and Requires CD28-Mediated Akt-Dependent and Independent Pathways. J. Immunol..

[94] Pollizzi K.N., Powell J.D. (2015). Regulation of T cells by mTOR: the known knowns and the known unknowns. Trends Immunol..

[95] Patsoukis N., Bardhan K., Chatterjee P., Sari D., Liu B., Bell L.N., Karoly E.D., Freeman G.J., Petkova V., Seth P. (2015). PD-1 alters T-cell metabolic reprogramming by inhibiting glycolysis and promoting lipolysis and fatty acid oxidation. Nat. Commun..

[96] Parry R.V., Chemnitz J.M., Frauwirth K.A., Lanfranco A.R., Braunstein I., Kobayashi S.V., Linsley P.S., Thompson C.B., Riley J.L. (2005). CTLA-4 and PD-1 Receptors Inhibit T-Cell Activation by Distinct Mechanisms. Mol. Cell. Biol..

[97] Patsoukis N., Li L., Sari D., Petkova V., Boussiotis V.A. (2013). PD-1 Increases PTEN Phosphatase Activity While Decreasing PTEN Protein Stability by Inhibiting Casein Kinase 2. Mol. Cell. Biol..

[98] Chang C.-H., Curtis J.D., Maggi L.B., Faubert B., Villarino A.V., O’Sullivan D., Huang S.C.-C., van der Windt G.J.W., Blagih J., Qiu J. (2013). Posttranscriptional control of T cell effector function by aerobic glycolysis. Cell.

[99] Zheng Y., Delgoffe G.M., Meyer C.F., Chan W., Powell J.D. (2009). Anergic T Cells Are Metabolically Anergic. J. Immunol..

[100] Nunes C., Wong R., Mason M., Fegan C., Man S., Pepper C. (2012). Expansion of a CD8(+)PD-1(+) replicative senescence phenotype in early stage CLL patients is associated with inverted CD4:CD8 ratios and disease progression. Clin. Cancer Res..

[101] Chen X., Liu S., Wang L., Zhang W.-G., Ji Y., Ma X. (2008). Clinical significance of B7-H1 (PD-L1) expression in human acute leukemia. Cancer Biol. Ther..

[102] Yang Z.-Z., Grote D.M., Xiu B., Ziesmer S.C., Price-Troska T.L., Hodge L.S., Yates D.M., Novak A.J., Ansell S.M. (2014). TGF-β upregulates CD70 expression and induces exhaustion of effector memory T cells in B-cell non-Hodgkin’s lymphoma. Leukemia.

[103] McClanahan F., Hanna B., Miller S., Clear A.J., Lichter P., Gribben J.G., Seiffert M. (2015). PD-L1 checkpoint blockade prevents immune dysfunction and leukemia development in a mouse model of chronic lymphocytic leukemia. Blood.

[104] Macintyre A.N., Gerriets V.A., Nichols A.G., Michalek R.D., Rudolph M.C., Deoliveira D., Anderson S.M., Abel E.D., Chen B.J., Hale L.P. (2014). The Glucose Transporter Glut1 Is Selectively Essential for CD4 T Cell Activation and Effector Function. Cell Metab..

[105] Michalek R.D., Gerriets V.A., Jacobs S.R., Macintyre A.N., MacIver N.J., Mason E.F., Sullivan S.A., Nichols A.G., Rathmell J.C. (2011). Cutting edge: distinct glycolytic and lipid oxidative metabolic programs are essential for effector and regulatory CD4+ T cell subsets. J. Immunol..

[106] Van Bruggen J.A.C., Martens A.W.J., Fraietta J.A., Hofland T., Tonino S.H., Eldering E., Levin M.-D., Siska P.J., Endstra S., Rathmell J.C. (2019). Chronic lymphocytic leukemia cells impair mitochondrial fitness in CD8+ T cells and impede CAR T-cell efficacy. Blood.

[107] Ron-Harel N., Santos D., Ghergurovich J.M., Sage P.T., Reddy A., Lovitch S.B., Dephoure N., Satterstrom F.K., Sheffer M., Spinelli J.B. (2016). Mitochondrial Biogenesis and Proteome Remodeling Promote One-Carbon Metabolism for T Cell Activation. Cell Metab..

[108] Fischer M., Bantug G.R., Dimeloe S., Gubser P.M., Burgener A.-V., Grählert J., Balmer M.L., Develioglu L., Steiner R., Unterstab G. (2018). Early effector maturation of naïve human CD8 ^+^ T cells requires mitochondrial biogenesis. Eur. J. Immunol..

[109] Van der Windt G.J.W., O’Sullivan D., Everts B., Huang S.C.-C., Buck M.D., Curtis J.D., Chang C.-H., Smith A.M., Ai T., Faubert B. (2013). CD8 memory T cells have a bioenergetic advantage that underlies their rapid recall ability. Proc. Natl. Acad. Sci. USA.

[110] Scharping N.E., Menk A.V., Moreci R.S., Whetstone R.D., Dadey R.E., Watkins S.C., Ferris R.L., Delgoffe G.M. (2016). The Tumor Microenvironment Represses T Cell Mitochondrial Biogenesis to Drive Intratumoral T Cell Metabolic Insufficiency and Dysfunction. Immunity.

[111] Lemos H., Huang L., Prendergast G.C., Mellor A.L. (2019). Immune control by amino acid catabolism during tumorigenesis and therapy. Nat. Rev. Cancer.

[112] Théate I., van Baren N., Pilotte L., Moulin P., Larrieu P., Renauld J.-C., Hervé C., Gutierrez-Roelens I., Marbaix E., Sempoux C. (2015). Extensive profiling of the expression of the indoleamine 2,3-dioxygenase 1 protein in normal and tumoral human tissues. Cancer Immunol. Res..

[113] El-Zaatari M., Bass A.J., Bowlby R., Zhang M., Syu L.-J., Yang Y., Grasberger H., Shreiner A., Tan B., Bishu S. (2018). Indoleamine 2,3-Dioxygenase 1, Increased in Human Gastric Pre-Neoplasia, Promotes Inflammation and Metaplasia in Mice and Is Associated With Type II Hypersensitivity/Autoimmunity. Gastroenterology.

[114] Prendergast G.C., Malachowski W.P., DuHadaway J.B., Muller A.J. (2017). Discovery of IDO1 Inhibitors: From Bench to Bedside. Cancer Res..

[115] Brandacher G., Perathoner A., Ladurner R., Schneeberger S., Obrist P., Winkler C., Werner E.R., Werner-Felmayer G., Weiss H.G., Göbel G. (2006). Prognostic Value of Indoleamine 2,3-Dioxygenase Expression in Colorectal Cancer: Effect on Tumor-Infiltrating T Cells. Clin. Cancer Res..

[116] Brody J.R., Costantino C.L., Berger A.C., Sato T., Lisanti M.P., Yeo C.J., Emmons R.V., Witkiewicz A.K. (2009). Expression of indoleamine 2,3-dioxygenase in metastatic malignant melanoma recruits regulatory T cells to avoid immune detection and affects survival. Cell Cycle.

[117] Corm S., Berthon C., Imbenotte M., Biggio V., Lhermitte M., Dupont C., Briche I., Quesnel B. (2009). Indoleamine 2,3-dioxygenase activity of acute myeloid leukemia cells can be measured from patients’ sera by HPLC and is inducible by IFN-gamma. Leuk. Res..

[118] Casero R.A., Murray Stewart T., Pegg A.E. (2018). Polyamine metabolism and cancer: treatments, challenges and opportunities. Nat. Rev. Cancer.

[119] Hesterberg R.S., Cleveland J.L., Epling-Burnette P.K. (2018). Role of Polyamines in Immune Cell Functions. Med. Sci..

[120] Vogel C.F.A., Goth S.R., Dong B., Pessah I.N., Matsumura F. (2008). Aryl hydrocarbon receptor signaling mediates expression of indoleamine 2,3-dioxygenase. Biochem. Biophys. Res. Commun..

[121] Mezrich J.D., Fechner J.H., Zhang X., Johnson B.P., Burlingham W.J., Bradfield C.A. (2010). An interaction between kynurenine and the aryl hydrocarbon receptor can generate regulatory T cells. J. Immunol..

[122] Nguyen N.T., Kimura A., Nakahama T., Chinen I., Masuda K., Nohara K., Fujii-Kuriyama Y., Kishimoto T. (2010). Aryl hydrocarbon receptor negatively regulates dendritic cell immunogenicity via a kynurenine-dependent mechanism. Proc. Natl. Acad. Sci. USA.

[123] Feng S., Cao Z., Wang X. (2013). Role of aryl hydrocarbon receptor in cancer. Biochim. Biophys. Acta-Rev. Cancer.

[124] Hayashi T., Mo J.-H., Gong X., Rossetto C., Jang A., Beck L., Elliott G.I., Kufareva I., Abagyan R., Broide D.H. (2007). 3-Hydroxyanthranilic acid inhibits PDK1 activation and suppresses experimental asthma by inducing T cell apoptosis. Proc. Natl. Acad. Sci. USA.

[125] Yan Y., Zhang G.-X., Gran B., Fallarino F., Yu S., Li H., Cullimore M.L., Rostami A., Xu H. (2010). IDO Upregulates Regulatory T Cells via Tryptophan Catabolite and Suppresses Encephalitogenic T Cell Responses in Experimental Autoimmune Encephalomyelitis. J. Immunol..

[126] Pallasch C.P., Ulbrich S., Brinker R., Hallek M., Uger R.A., Wendtner C.-M. (2009). Disruption of T cell suppression in chronic lymphocytic leukemia by CD200 blockade. Leuk. Res..

[127] Wang W., Huang L., Jin J.-Y., Jolly S., Zang Y., Wu H., Yan L., Pi W., Li L., Mellor A.L. (2018). IDO Immune Status after Chemoradiation May Predict Survival in Lung Cancer Patients. Cancer Res..

[128] Muller A.J., DuHadaway J.B., Donover P.S., Sutanto-Ward E., Prendergast G.C. (2005). Inhibition of indoleamine 2,3-dioxygenase, an immunoregulatory target of the cancer suppression gene Bin1, potentiates cancer chemotherapy. Nat. Med..

[129] Hou D.-Y., Muller A.J., Sharma M.D., DuHadaway J., Banerjee T., Johnson M., Mellor A.L., Prendergast G.C., Munn D.H. (2007). Inhibition of Indoleamine 2,3-Dioxygenase in Dendritic Cells by Stereoisomers of 1-Methyl-Tryptophan Correlates with Antitumor Responses. Cancer Res..

[130] Triplett T.A., Garrison K.C., Marshall N., Donkor M., Blazeck J., Lamb C., Qerqez A., Dekker J.D., Tanno Y., Lu W.-C. (2018). Reversal of indoleamine 2,3-dioxygenase-mediated cancer immune suppression by systemic kynurenine depletion with a therapeutic enzyme. Nat. Biotechnol..

[131] Banerjee T., Duhadaway J.B., Gaspari P., Sutanto-Ward E., Munn D.H., Mellor A.L., Malachowski W.P., Prendergast G.C., Muller A.J. (2008). A key in vivo antitumor mechanism of action of natural product-based brassinins is inhibition of indoleamine 2,3-dioxygenase. Oncogene.

[132] Choe J.-Y., Yun J.Y., Jeon Y.K., Kim S.H., Park G., Huh J.R., Oh S., Kim J.E. (2014). Indoleamine 2,3-dioxygenase (IDO) is frequently expressed in stromal cells of Hodgkin lymphoma and is associated with adverse clinical features: a retrospective cohort study. BMC Cancer.

[133] Pötzl J., Roser D., Bankel L., Hömberg N., Geishauser A., Brenner C.D., Weigand M., Röcken M., Mocikat R. (2017). Reversal of tumor acidosis by systemic buffering reactivates NK cells to express IFN-γ and induces NK cell-dependent lymphoma control without other immunotherapies. Int. J. cancer.

[134] Lacroix R., Rozeman E.A., Kreutz M., Renner K., Blank C.U. (2018). Targeting tumor-associated acidity in cancer immunotherapy. Cancer Immunol. Immunother..

[135] Pilon-Thomas S., Kodumudi K.N., El-Kenawi A.E., Russell S., Weber A.M., Luddy K., Damaghi M., Wojtkowiak J.W., Mulé J.J., Ibrahim-Hashim A. (2016). Neutralization of Tumor Acidity Improves Antitumor Responses to Immunotherapy. Cancer Res..

[136] Thorens B., Mueckler M. (2010). Glucose transporters in the 21st Century. Am. J. Physiol. Endocrinol. Metab..

[137] Gonzalez P.S., O’Prey J., Cardaci S., Barthet V.J.A., Sakamaki J., Beaumatin F., Roseweir A., Gay D.M., Mackay G., Malviya G. (2018). Mannose impairs tumour growth and enhances chemotherapy. Nature.

[138] Murata H., Hruz P.W., Mueckler M. (2000). The Mechanism of Insulin Resistance Caused by HIV Protease Inhibitor Therapy. J. Biol. Chem..

[139] Adekola K.U.A., Dalva Aydemir S., Ma S., Zhou Z., Rosen S.T., Shanmugam M. (2015). Investigating and targeting chronic lymphocytic leukemia metabolism with the human immunodeficiency virus protease inhibitor ritonavir and metformin. Leuk. Lymphoma.

[140] Dalva-Aydemir S., Bajpai R., Martinez M., Adekola K.U.A., Kandela I., Wei C., Singhal S., Koblinski J.E., Raje N.S., Rosen S.T. (2015). Targeting the metabolic plasticity of multiple myeloma with FDA-approved ritonavir and metformin. Clin. Cancer Res..

[141] Eikawa S., Nishida M., Mizukami S., Yamazaki C., Nakayama E., Udono H. (2015). Immune-mediated antitumor effect by type 2 diabetes drug, metformin. Proc. Natl. Acad. Sci. USA.

[142] Cha J.-H., Yang W.-H., Xia W., Wei Y., Chan L.-C., Lim S.-O., Li C.-W., Kim T., Chang S.-S., Lee H.-H. (2018). Metformin Promotes Antitumor Immunity via Endoplasmic-Reticulum-Associated Degradation of PD-L1. Mol. Cell.

[143] Pereira F.V., Melo A.C.L., Low J.S., de Castro Í.A., Braga T.T., Almeida D.C., Batista de Lima A.G.U., Hiyane M.I., Correa-Costa M., Andrade-Oliveira V. (2018). Metformin exerts antitumor activity via induction of multiple death pathways in tumor cells and activation of a protective immune response. Oncotarget.

[144] Kunisada Y., Eikawa S., Tomonobu N., Domae S., Uehara T., Hori S., Furusawa Y., Hase K., Sasaki A., Udono H. (2017). Attenuation of CD4+CD25+ Regulatory T Cells in the Tumor Microenvironment by Metformin, a Type 2 Diabetes Drug. EBioMedicine.

[145] Wang F., Travins J., DeLaBarre B., Penard-Lacronique V., Schalm S., Hansen E., Straley K., Kernytsky A., Liu W., Gliser C. (2013). Targeted Inhibition of Mutant IDH2 in Leukemia Cells Induces Cellular Differentiation. Science.

[146] Chaturvedi A., Herbst L., Pusch S., Klett L., Goparaju R., Stichel D., Kaulfuss S., Panknin O., Zimmermann K., Toschi L. (2017). Pan-mutant-IDH1 inhibitor BAY1436032 is highly effective against human IDH1 mutant acute myeloid leukemia in vivo. Leukemia.

[147] Fruman D.A., Rommel C. (2011). PI3K Inhibitors in Cancer: Rationale and Serendipity Merge in the Clinic. Cancer Discov..

[148] Gopal A.K., Kahl B.S., de Vos S., Wagner-Johnston N.D., Schuster S.J., Jurczak W.J., Flinn I.W., Flowers C.R., Martin P., Viardot A. (2014). PI3Kδ Inhibition by Idelalisib in Patients with Relapsed Indolent Lymphoma. N. Engl. J. Med..

[149] Brown J.R., Byrd J.C., Coutre S.E., Benson D.M., Flinn I.W., Wagner-Johnston N.D., Spurgeon S.E., Kahl B.S., Bello C., Webb H.K. (2014). Idelalisib, an inhibitor of phosphatidylinositol 3-kinase p110, for relapsed/refractory chronic lymphocytic leukemia. Blood.

[150] Ali K., Soond D.R., Pineiro R., Hagemann T., Pearce W., Lim E.L., Bouabe H., Scudamore C.L., Hancox T., Maecker H. (2014). Inactivation of PI(3)K p110δ breaks regulatory T-cell-mediated immune tolerance to cancer. Nature.

[151] Da Roit F., Engelberts P.J., Taylor R.P., Breij E.C.W., Gritti G., Rambaldi A., Introna M., Parren P.W.H.I., Beurskens F.J., Golay J. (2015). Ibrutinib interferes with the cell-mediated anti-tumor activities of therapeutic CD20 antibodies: implications for combination therapy. Haematologica.

[152] Druker B.J., Talpaz M., Resta D.J., Peng B., Buchdunger E., Ford J.M., Lydon N.B., Kantarjian H., Capdeville R., Ohno-Jones S. (2001). Efficacy and safety of a specific inhibitor of the BCR-ABL tyrosine kinase in chronic myeloid leukemia. N. Engl. J. Med..

[153] Rea D., Etienne G., Nicolini F., Cony-Makhoul P., Johnson-Ansah H., Legros L., Huguet F., Tulliez M., Gardembas M., Bouabdallah K. (2012). First-line imatinib mesylate in patients with newly diagnosed accelerated phase-chronic myeloid leukemia. Leukemia.

[154] Gottschalk S., Anderson N., Hainz C., Eckhardt S.G., Serkova N.J. (2004). Imatinib (STI571)-mediated changes in glucose metabolism in human leukemia BCR-ABL-positive cells. Clin. Cancer Res..

[155] Barnes K., McIntosh E., Whetton A.D., Daley G.Q., Bentley J., Baldwin S.A. (2005). Chronic myeloid leukaemia: an investigation into the role of Bcr-Abl-induced abnormalities in glucose transport regulation. Oncogene.

[156] Bilban M., Heintel D., Scharl T., Woelfel T., Auer M.M., Porpaczy E., Kainz B., Kröber A., Carey V.J., Shehata M. (2006). Deregulated expression of fat and muscle genes in B-cell chronic lymphocytic leukemia with high lipoprotein lipase expression. Leukemia.

[157] Galicia-Vázquez G., Smith S., Aloyz R. (2018). Del11q-positive CLL lymphocytes exhibit altered glutamine metabolism and differential response to GLS1 and glucose metabolism inhibition. Blood Cancer J..

[158] Long M., Beckwith K., Do P., Mundy B.L., Gordon A., Lehman A.M., Maddocks K.J., Cheney C., Jones J.A., Flynn J.M. (2017). Ibrutinib treatment improves T cell number and function in CLL patients. J. Clin. Investig..

[159] Dubovsky J.A., Beckwith K.A., Natarajan G., Woyach J.A., Jaglowski S., Zhong Y., Hessler J.D., Liu T.-M., Chang B.Y., Larkin K.M. (2013). Ibrutinib is an irreversible molecular inhibitor of ITK driving a Th1-selective pressure in T lymphocytes. Blood.

[160] Natarajan G., Terrazas C., Oghumu S., Varikuti S., Dubovsky J.A., Byrd J.C., Satoskar A.R. (2016). Ibrutinib enhances IL-17 response by modulating the function of bone marrow derived dendritic cells. Oncoimmunology.

[161] Ruella M., Kenderian S.S., Shestova O., Fraietta J.A., Qayyum S., Zhang Q., Maus M.V., Liu X., Nunez-Cruz S., Klichinsky M. (2016). The Addition of the BTK Inhibitor Ibrutinib to Anti-CD19 Chimeric Antigen Receptor T Cells (CART19) Improves Responses against Mantle Cell Lymphoma. Clin. Cancer Res..

[162] Gunderson A.J., Kaneda M.M., Tsujikawa T., Nguyen A.V., Affara N.I., Ruffell B., Gorjestani S., Liudahl S.M., Truitt M., Olson P. (2016). Bruton Tyrosine Kinase-Dependent Immune Cell Cross-talk Drives Pancreas Cancer. Cancer Discov..

[163] Skarzynski M., Niemann C.U., Lee Y.S., Martyr S., Maric I., Salem D., Stetler-Stevenson M., Marti G.E., Calvo K.R., Yuan C. (2016). Interactions between Ibrutinib and Anti-CD20 Antibodies: Competing Effects on the Outcome of Combination Therapy. Clin. Cancer Res..

[164] Pallasch C.P., Schwamb J., Königs S., Schulz A., Debey S., Kofler D., Schultze J.L., Hallek M., Ultsch A., Wendtner C.-M. (2008). Targeting lipid metabolism by the lipoprotein lipase inhibitor orlistat results in apoptosis of B-cell chronic lymphocytic leukemia cells. Leukemia.

[165] Woyach J.A. (2017). How I manage ibrutinib-refractory chronic lymphocytic leukemia. Blood.

[166] Rozovski U., Harris D.M., Li P., Liu Z., Jain P., Ferrajoli A., Burger J., Thompson P., Jain N., Wierda W. (2018). Ibrutinib inhibits free fatty acid metabolism in chronic lymphocytic leukemia. Leuk. Lymphoma.

[167] Warburg O. (1956). On the Origin of Cancer Cells. Science.

[168] Jones C.L., Stevens B.M., D’Alessandro A., Culp-Hill R., Reisz J.A., Pei S., Gustafson A., Khan N., DeGregori J., Pollyea D.A. (2019). Cysteine depletion targets leukemia stem cells through inhibition of electron transport complex II. Blood.

[169] Jones C.L., Stevens B.M., D’Alessandro A., Reisz J.A., Culp-Hill R., Nemkov T., Pei S., Khan N., Adane B., Ye H. (2018). Inhibition of Amino Acid Metabolism Selectively Targets Human Leukemia Stem Cells. Cancer Cell.

[170] Asselin B.L. (1999). The three asparaginases. Comparative pharmacology and optimal use in childhood leukemia. Adv. Exp. Med. Biol..

[171] Silverman L.B., Supko J.G., Stevenson K.E., Woodward C., Vrooman L.M., Neuberg D.S., Asselin B.L., Athale U.H., Clavell L., Cole P.D. (2010). Intravenous PEG-asparaginase during remission induction in children and adolescents with newly diagnosed acute lymphoblastic leukemia. Blood.

[172] Som A., Raliya R., Tian L., Akers W., Ippolito J.E., Singamaneni S., Biswas P., Achilefu S. (2016). Monodispersed calcium carbonate nanoparticles modulate local pH and inhibit tumor growth in vivo. Nanoscale.

[173] Howell J.J., Hellberg K., Turner M., Talbott G., Kolar M.J., Ross D.S., Hoxhaj G., Saghatelian A., Shaw R.J., Manning B.D. (2017). Metformin Inhibits Hepatic mTORC1 Signaling via Dose-Dependent Mechanisms Involving AMPK and the TSC Complex. Cell Metab..

[174] Elstrom R.L., Bauer D.E., Buzzai M., Karnauskas R., Harris M.H., Plas D.R., Zhuang H., Cinalli R.M., Alavi A., Rudin C.M. (2004). Akt stimulates aerobic glycolysis in cancer cells. Cancer Res..

[175] Jager S., Handschin C., St.-Pierre J., Spiegelman B.M. (2007). AMP-activated protein kinase (AMPK) action in skeletal muscle via direct phosphorylation of PGC-1. Proc. Natl. Acad. Sci. USA.

[176] Coloff J.L., Macintyre A.N., Nichols A.G., Liu T., Gallo C.A., Plas D.R., Rathmell J.C. (2011). Akt-dependent glucose metabolism promotes Mcl-1 synthesis to maintain cell survival and resistance to Bcl-2 inhibition. Cancer Res.

[177] Kurelac I., Umesh Ganesh N., Iorio M., Porcelli A.M., Gasparre G. (2019). The multifaceted effects of metformin on tumor microenvironment. Semin. Cell Dev. Biol..

[178] Ley T.J., Miller C., Ding L., Raphael B.J., Mungall A.J., Robertson A.G., Hoadley K., Triche T.J., Laird P.W., Cancer Genome Atlas Research Network (2013). Genomic and epigenomic landscapes of adult de novo acute myeloid leukemia. N. Engl. J. Med..

[179] Losman J.-A., Looper R.E., Koivunen P., Lee S., Schneider R.K., McMahon C., Cowley G.S., Root D.E., Ebert B.L., Kaelin W.G. (2013). (R)-2-Hydroxyglutarate Is Sufficient to Promote Leukemogenesis and Its Effects Are Reversible. Science.

[180] Pusch S., Krausert S., Fischer V., Balss J., Ott M., Schrimpf D., Capper D., Sahm F., Eisel J., Beck A.-C. (2017). Pan-mutant IDH1 inhibitor BAY 1436032 for effective treatment of IDH1 mutant astrocytoma in vivo. Acta Neuropathol..

[181] Locatelli S.L., Careddu G., Serio S., Consonni F.M., Maeda A., Viswanadha S., Vakkalanka S., Castagna L., Santoro A., Allavena P. (2019). Targeting Cancer Cells and Tumor Microenvironment in Preclinical and Clinical Models of Hodgkin Lymphoma Using the Dual PI3Kdelta/gamma Inhibitor RP6530. Clin Cancer Res.

[182] De Rosa V., Monti M., Terlizzi C., Fonti R., Del Vecchio S., Iommelli F. (2019). Coordinate Modulation of Glycolytic Enzymes and OXPHOS by Imatinib in BCR-ABL Driven Chronic Myelogenous Leukemia Cells. Int. J. Mol. Sci..

[183] Shinohara H., Kumazaki M., Minami Y., Ito Y., Sugito N., Kuranaga Y., Taniguchi K., Yamada N., Otsuki Y., Naoe T. (2016). Perturbation of energy metabolism by fatty-acid derivative AIC-47 and imatinib in BCR-ABL-harboring leukemic cells. Cancer Lett..

[184] Buzzai M., Bauer D.E., Jones R.G., Deberardinis R.J., Hatzivassiliou G., Elstrom R.L., Thompson C.B. (2005). The glucose dependence of Akt-transformed cells can be reversed by pharmacologic activation of fatty acid beta-oxidation. Oncogene.

[185] Jeon S.-M., Chandel N.S., Hay N. (2012). AMPK regulates NADPH homeostasis to promote tumour cell survival during energy stress. Nature.

[186] Samudio I., Harmancey R., Fiegl M., Kantarjian H., Konopleva M., Korchin B., Kaluarachchi K., Bornmann W., Duvvuri S., Taegtmeyer H. (2010). Pharmacologic inhibition of fatty acid oxidation sensitizes human leukemia cells to apoptosis induction. J. Clin. Investig..

[187] Shinohara H., Sugito N., Kuranaga Y., Heishima K., Minami Y., Naoe T., Akao Y. (2019). Potent antiproliferative effect of fatty-acid derivative AIC -47 on leukemic mice harboring BCR—ABL mutation. Cancer Sci..

[188] Ito K., Carracedo A., Weiss D., Arai F., Ala U., Avigan D.E., Schafer Z.T., Evans R.M., Suda T., Lee C.-H. (2012). A PML–PPAR-δ pathway for fatty acid oxidation regulates hematopoietic stem cell maintenance. Nat. Med..

[189] Tili E., Michaille J.-J., Luo Z., Volinia S., Rassenti L.Z., Kipps T.J., Croce C.M. (2012). The down-regulation of miR-125b in chronic lymphocytic leukemias leads to metabolic adaptation of cells to a transformed state. Blood.

[190] Frank D.A., Mahajan S., Ritz J. (1997). B lymphocytes from patients with chronic lymphocytic leukemia contain signal transducer and activator of transcription (STAT) 1 and STAT3 constitutively phosphorylated on serine residues. J. Clin. Investig..

[191] Goldberg I.J. (1996). Lipoprotein lipase and lipolysis: central roles in lipoprotein metabolism and atherogenesis. J. Lipid Res..

[192] Kersten S. (2014). Integrated physiology and systems biology of PPARα. Mol. Metab..

[193] Oppezzo P., Vasconcelos Y., Settegrana C., Jeannel D., Vuillier F., Legarff-Tavernier M., Kimura E.Y., Bechet S., Dumas G., Brissard M. (2005). The LPL/ADAM29 expression ratio is a novel prognosis indicator in chronic lymphocytic leukemia. Blood.

[194] Zhang L., Yao Y., Zhang S., Liu Y., Guo H., Ahmed M., Bell T., Zhang H., Han G., Lorence E. (2019). Metabolic reprogramming toward oxidative phosphorylation identifies a therapeutic target for mantle cell lymphoma. Sci. Transl. Med..

[195] Nicklin P., Bergman P., Zhang B., Triantafellow E., Wang H., Nyfeler B., Yang H., Hild M., Kung C., Wilson C. (2009). Bidirectional transport of amino acids regulates mTOR and autophagy. Cell.

[196] Galicia-Vázquez G., Aloyz R. (2018). Ibrutinib Resistance Is Reduced by an Inhibitor of Fatty Acid Oxidation in Primary CLL Lymphocytes. Front. Oncol..

[197] Mayer R.L., Schwarzmeier J.D., Gerner M.C., Bileck A., Mader J.C., Meier-Menches S.M., Gerner S.M., Schmetterer K.G., Pukrop T., Reichle A. (2018). Proteomics and metabolomics identify molecular mechanisms of aging potentially predisposing for chronic lymphocytic leukemia. Mol. Cell. Proteomics.

[198] Divakaruni A.S., Hsieh W.Y., Minarrieta L., Duong T.N., Kim K.K.O., Desousa B.R., Andreyev A.Y., Bowman C.E., Caradonna K., Dranka B.P. (2018). Etomoxir Inhibits Macrophage Polarization by Disrupting CoA Homeostasis. Cell Metab..

[199] Jain P., Javdan M., Feger F.K., Chiu P.Y., Sison C., Damle R.N., Bhuiya T.A., Sen F., Abruzzo L.V., Burger J.A. (2012). Th17 and non-Th17 interleukin-17-expressing cells in chronic lymphocytic leukemia: delineation, distribution, and clinical relevance. Haematologica.

[200] Sagiv-Barfi I., Kohrt H.E.K., Czerwinski D.K., Ng P.P., Chang B.Y., Levy R. (2015). Therapeutic antitumor immunity by checkpoint blockade is enhanced by ibrutinib, an inhibitor of both BTK and ITK. Proc. Natl. Acad. Sci. USA.

[201] Sagiv-Barfi I., Kohrt H.E., Burckhardt L., Czerwinski D.K., Levy R. (2015). Ibrutinib enhances the antitumor immune response induced by intratumoral injection of a TLR9 ligand in mouse lymphoma. Blood.

[202] Papin A., Tessoulin B., Bellanger C., Moreau A., Le Bris Y., Maisonneuve H., Moreau P., Touzeau C., Amiot M., Pellat-Deceunynck C. (2019). CSF1R and BTK inhibitions as novel strategies to disrupt the dialog between mantle cell lymphoma and macrophages. Leukemia.

[203] Kohrt H.E., Sagiv-Barfi I., Rafiq S., Herman S.E.M., Butchar J.P., Cheney C., Zhang X., Buggy J.J., Muthusamy N., Levy R. (2014). Ibrutinib antagonizes rituximab-dependent NK cell-mediated cytotoxicity. Blood.

[204] Zub K.A., Sousa M.M., Sarno A., Sharma A., Demirovic A., Rao S., Young C., Aas P.A., Ericsson I., Sundan A. (2015). Modulation of cell metabolic pathways and oxidative stress signaling contribute to acquired melphalan resistance in multiple myeloma cells. PLoS One.

[205] Pollyea D.A., Stevens B.M., Jones C.L., Winters A., Pei S., Minhajuddin M., D’Alessandro A., Culp-Hill R., Riemondy K.A., Gillen A.E. (2018). Venetoclax with azacitidine disrupts energy metabolism and targets leukemia stem cells in patients with acute myeloid leukemia. Nat. Med..

[206] Herling C.D., Abedpour N., Weiss J., Schmitt A., Jachimowicz R.D., Merkel O., Cartolano M., Oberbeck S., Mayer P., Berg V. (2018). Clonal dynamics towards the development of venetoclax resistance in chronic lymphocytic leukemia. Nat. Commun..

[207] Pui C.-H., Evans W.E. (2006). Treatment of Acute Lymphoblastic Leukemia. N. Engl. J. Med..

[208] Heo Y.-A., Syed Y.Y., Keam S.J. (2019). Pegaspargase: A Review in Acute Lymphoblastic Leukaemia. Drugs.

